# Haplotypes across the Oceans: worldwide phylogeography, evolution, conservation and nomenclature standardization in the leatherback sea turtle (*Dermochelys coriacea*)

**DOI:** 10.1371/journal.pone.0354151

**Published:** 2026-08-19

**Authors:** Wesley D. Colombo, Sarah de S. A. Teodoro, João Luiz G. da Fonseca, Gabrielly L. Schultz, Sarah M. Vargas

**Affiliations:** 1 Departamento de Zoologia, Universidade Federal de Minas Gerais, Belo Horizonte, Minas Gerais, Brazil; 2 Departamento de Ciências Biológicas, Universidade Federal do Espírito Santo, Vitória, Espírito Santo, Brazil; State Museum of Natural History, GERMANY

## Abstract

Inconsistent haplotype nomenclature complicates the integration of genetic data in population genetics and phylogeographic studies of the leatherback sea turtle (*Dermochelys coriacea*). Previous studies using control region mitochondrial DNA (mtDNA) sequences of varying lengths (e.g., 496 bp, 711 bp, 763 bp) have led to redundant classifications and misinterpretations of population structure. In this study, we reviewed 304 control region mtDNA sequences from GenBank, representing all published haplotypes across the Atlantic, Indian, and Pacific Oceans. We proposed a standardized nomenclature based on two sequence lengths: 473 bp (short) and 681 bp (long). We identified 32 haplotypes in the long-sequence dataset and 24 in the short-sequence dataset, with 21 and 14 variable sites, respectively. Haplotype network analyses revealed strong genetic structure, with Dc1.1 (long) and Dc1 (short) as the most widely distributed haplotypes. Ancestral area reconstruction indicated a Pacific origin for the most basal *D. coriacea* nodes, followed by multiple transitions into the Atlantic and Indian Oceans. Divergence time estimates placed the origin of the *D. coriacea* contemporary clade at ~4.86 million years ago (Ma), with Dc1.1 emerging around 0.49 Ma. Atlantic colonizations occurred at ~1.75 Ma and ~1.73 Ma, and Indian Ocean colonization at ~3.00 Ma. These findings support the adoption of a unified haplotype nomenclature to clarify population genetic structure and enhance the consistency of genetic assessments for conservation applications.

## Introduction

The leatherback turtle, *Dermochelys coriacea* (Vandelli, 1761), is the largest extant sea turtle species and a keystone species in marine ecosystems. Its global distribution spans tropical and subtropical oceans, and the species diverged from other sea turtles during the Jurassic and Cretaceous periods, approximately 100–150 million years ago [1,2]. This ancient lineage is characterized by high behavioral plasticity, enabling diverse habitat use and foraging strategies [3].

Globally, leatherback sea turtle populations are organized into seven Regional Management Units (RMUs), which encompass nine mitochondrial DNA (mtDNA) stocks, and ~652 rookeries distributed across the Pacific, Atlantic, and Indian Oceans [4–6]. These turtles exhibit complex migratory behaviors, traveling vast distances between nesting beaches and feeding areas [7]. Genetic studies have predominantly focused on nesting populations, providing valuable insights into phylogeography and population connectivity [7–14]. Key investigations have covered the Atlantic [7,9,10,12,14], Pacific [8,10,15], and Indo-Pacific Oceans [7,13,16–18], offering critical insights into the genetic diversity and conservation needs of the species.

In addition to nesting areas, leatherback turtles are frequently encountered in coastal and offshore waters, where individuals are often found stranded, caught as bycatch, or feeding. However, the origins of these individuals, particularly those forming non-nesting aggregations, remain poorly understood [7]. Studies analyzing turtle aggregations, excluding nesting sites, have been conducted in the Atlantic [14,19–22] and the Pacific [23]. Despite these efforts, such studies are still limited in scope and geographic coverage, leaving significant gaps in knowledge about population connectivity and migration dynamics.

Early genetic studies [8,10] utilized mtDNA control region (*D-loop*) sequences of 496 base pairs (bp), establishing the foundation for understanding global population connectivity. These studies identified key haplotypes associated with major rookeries, such as those in the Northwest Atlantic, Southeast Caribbean, and Western Pacific. A nomenclature system was proposed based on alphabetical designations (e.g., A–L) and the identification of 11 haplotypes [10]. Subsequent research expanded upon these findings, revealing seven haplotypes (Dc1–Dc7) in the Southwest Atlantic using longer sequences (~711 bp) [14]. The nomenclature was further refined, with 10 haplotypes identified (e.g., Dc1.1, Dc1.3, Dc1.4) using sequences extending to 763 bp, while also attempting to reconcile nomenclatures across shorter and longer sequences [9].

Despite these advances, sequence length and nomenclature inconsistencies have posed significant challenges for data integration and cross-study comparisons. Similar issues have been documented in other sea turtle species, where efforts have been made to reconcile haplotype nomenclature across datasets of varying sequence lengths and to improve standardization through collaborative approaches. For example, studies on green turtles (*Chelonia mydas*) have highlighted the importance of comprehensive phylogeographic frameworks and consistent haplotype naming conventions [24,25], while work on loggerhead turtles (*Caretta caretta*) has demonstrated the value of coordinated working groups in integrating short and long mtDNA sequences into unified nomenclature systems [26]. In leatherbacks, for example, three haplotypes (JD1–JD3) from Pacific were identified using sequences of 861 bp [23], while three Indo-Pacific haplotypes (Hap1–Hap3) were cited based on variable sequence lengths ranging from 644 to 757 bp [13]. Furthermore, six unpublished sequences deposited in GenBank highlight additional Pacific haplotypes (763 bp) that have not yet been incorporated into comparative frameworks [17,27]. Nomenclature differences in short haplotypes, such as using numeric codes [17] instead of letters [10], add to the complexity and highlight the need for standardization to enable consistent comparisons.

The discrepancies in sequence lengths are particularly problematic in studies of haplotypes, where even a single base pair can influence the resolution of population structure inferences. Longer sequences (~711 bp or longer) have been shown to capture greater genetic variation and resolve subtle differences among populations. For instance, it was demonstrated that longer sequences allowed the differentiation of haplotypes (e.g., Dc1.1, Dc1.3, Dc1.4) previously grouped within haplotype A [9]. Recent studies have emphasized harmonizing sequence lengths and nomenclature systems. Longer sequences were employed to uncover previously undetected diversity in the Andaman Sea [13] and Western Pacific [23], underscoring the value of consistent methodologies in facilitating global conservation efforts.

To address these issues, this study proposes a standardized haplotype nomenclature for *D. coriacea* based on 473 bp (short) mtDNA sequences and 681 bp (long). This unified approach incorporates historical data and sequences from key rookeries and foraging areas. Additionally, the ancestral origins of these haplotypes are inferred, investigating dispersal events and their distribution over evolutionary timescales. This effort seeks to support global conservation strategies for this highly migratory and vulnerable species by providing a robust phylogeographic framework.

## Materials and methods

### Mitochondrial DNA sequences

We reviewed all mtDNA sequences deposited in GenBank for the control region of *D. coriacea* publicly available up to 1 February 2026. A total of 304 sequences corresponding to all known haplotypes reported for different regions (Atlantic, Indian, and Pacific Oceans), as follows: one sequence (496 bp) corresponding to haplotype A (GenBank accession number AF121964) [10]; for the remaining ten haplotypes (B–I, K–L), we replicated the sequence for haplotype A and introduced nucleotide substitutions as described by [10]; seven sequences (711 bp) (GenBank accession numbers EF513272–EF513278), corresponding to the seven haplotypes published along the Brazilian coast [14]; eight sequences (763 bp) corresponding to haplotypes identified from Pacific leatherback turtles (GenBank accession numbers HM452354, HM452358, HM452359, HM452360, HM452361, HM452363, HM452364, and PQ285430) [17]; six sequences (763 bp) corresponding to haplotypes identified from Pacific leatherback turtles (GenBank accession numbers HM452353, HM452355––HM452357, HM452362, and HM452365), which remain unpublished and have not been analyzed in haplotype networks [27]; four sequences ranging from 862–982 bp (GenBank accession numbers JX454969, JX454973, JX454989, and JX454992) were obtained from genomic data [28]; ten sequences (763 bp) corresponding to haplotypes published by [9] (GenBank accession numbers HM452343–HM452352); 68 sequences (711 bp) corresponding to five haplotypes identified by [12] (GenBank accession numbers JX629672–JX629739); 16 sequences (861 bp) from [23] (GenBank accession numbers LC159496–LC159511); one sequence (993 bp) from [29] (GenBank accession number MF460363); 13 sequences (695 bp) published by [7] (GenBank accession numbers MF346872–MF346884); two sequences (588 bp) from [13,19] (GenBank accession numbers MK674797 and MK674798); 153 sequences (644–757 bp) published by [13] (GenBank accession numbers OK040245–OK040397). Of these, nine sequences (OK040389–OK040397) were not cited in the publication and lacked defined haplotypes but were included as they were deposited in GenBank; one sequence (763 bp) from [20] (GenBank accession number OQ621749); three sequences (732 bp) from [15] (GenBank accession numbers OP716916–OP716918); one sequence (763 bp) from Sumatra (GenBank accession number PX561101). Two additional sequences (745 bp) published by [15], corresponding to haplotypes H1 and H5, were excluded from the analysis because they were identified as nuclear mitochondrial DNA segments (NUMTs) [30]. Finally, two sequences, corresponding to haplotypes 4.2 (GenBank accession number KU234548) and 4.3 (GenBank accession number KU234549), were excluded from the analysis because they were identified as sequencing errors [18].

### Nomenclature of haplotypes

We standardized the nomenclature of haplotypes using two alignments: one based on short sequences (473 bp) and another on long sequences (681 bp) from the mtDNA control region. Short sequences were aligned with the sequence of haplotype A (GenBank accession number AF121964) to identify the positions of the nine polymorphisms described for 496 bp sequences [10]. During preprocessing, the first 23 bp of the sequences were removed to eliminate regions without detected polymorphisms and to standardize the sequence length across all samples. To standardize haplotype nomenclature for short sequences, we adopted the prefix “Dc”, as previously proposed [9], combined with the numerical system introduced by [17,18]. For instance, haplotype “A” from [10] is now referred to as “Dc1”.

Long sequences were aligned with the sequence of haplotype Dc1.1 (GenBank accession number HM452343) to identify the positions of the ten polymorphisms described for 763 bp sequences [9]. A total of 271 sequences were used, excluding all 12 sequences from [10], 17 sequences from [13] (GenBank accession numbers OK040253, OK040264, OK040265, OK040277, OK040297, OK040306, OK040319, OK040375, OK040389–OK040397), and both sequences from [19], as they correspond to short or poor-quality sequence regions. Retaining them in the long-sequence alignment would have required a more extensive truncation. All sequences used in the long-sequence alignment were further trimmed by removing the first 14 bp, as no polymorphisms were detected in this region. We standardized the naming system based on [9] for nomenclature of long-sequence haplotypes, using a numeric designation after the prefix “Dc”.

To maintain consistency between datasets, long haplotypes were named using a hierarchical system based on the corresponding short haplotype. Short haplotypes retained the format DcX, whereas long haplotypes were designated as DcX.Y, where X indicates the associated short haplotype and Y identifies distinct variants detected in the longer alignment. Under this system, multiple long haplotypes may correspond to the same short haplotype but differ due to additional polymorphisms revealed in the longer sequence (e.g., Dc1.1, Dc1.2, Dc1.3 all correspond to the short haplotype Dc1).

### Haplotype networks

A haplotype network was constructed using the Median Joining method (95% confidence level) in PopART v1.7 software [31] and subsequently edited in an image editor. Two separate networks were generated: one based on long-sequence haplotypes and another on short-sequence haplotypes. To ensure consistency and improve the representation of haplotype diversity, longer sequences retrieved from GenBank were aligned and trimmed to match the short-sequence dataset. This adjustment facilitated a more comprehensive comparison of haplotypes while preserving dataset integrity.

The networks were visualized to assess genetic relationships among haplotypes across different oceanic regions (Atlantic, Indian, and Pacific Oceans). Since not all studies with sequences deposited in GenBank provided haplotype frequency data, the networks were constructed solely based on the presence or absence of each haplotype in each locality (S1 Table). This approach ensured dataset consistency and enabled a standardized representation of genetic relationships among haplotypes across different oceanic regions.

### Ancestral area reconstruction and divergence time analyses

An alignment of 740 bp and 46 control region sequences was used to perform a Bayesian Inference (BI), which supplied the basis for divergence times, evolutionary rates, and ancestral area reconstruction. This alignment included two Chelydridae species as outgroups: *Chelydra serpentina* (GenBank accession number NC_011198) and *Macrochelys temminckii* (GenBank accession number NC_009260). Additionally, twelve sequences from Cheloniidae species were included: two from *Caretta caretta* (GenBank accession numbers KC748470 and NC_016923), two from *Chelonia mydas* (GenBank accession numbers KF311762 and ON791800), two from *Eretmochelys imbricata* (GenBank accession numbers NC_012398 and PP826981), two from *Lepidochelys kempii* (GenBank accession numbers MN159143 and MN159152), two from *L. olivacea* (GenBank accession numbers KY091852 and NC_028634), and two from *Natator depressus* (GenBank accession numbers MN029088 and MN029107). The tree was rooted with the outgroup common snapping turtle (*C. serpentina*) and alligator snapping turtle (*M. temminckii*), following the methodology previously suggested [28]. The remaining sequences correspond to all 32 known *D. coriacea* haplotypes.

The BI analysis was conducted using BEAST v2.7.7 [32]. The analysis was performed with a chain length of 50,000,000 generations, sampling every 1,000 steps. Convergence and adequate effective sample size (ESS) values were inspected in Tracer v1.7.2 [33] to assess Markov chain Monte Carlo (MCMC) mixing and confirm convergence. We used the bModelTest package [34] implemented in BEAST v2.7.7. This approach allows for Bayesian model averaging over a range of reversible nucleotide substitution models using reversible jump Markov chain Monte Carlo (rjMCMC) [35]. The substitution model with the most posterior support was 123343 and 121131 with 40.91% of cumulative support. We used a relaxed log-normal clock model [36] implemented in BEAST v2.7.7 to estimate divergence times and evolutionary rates with greater reliability. In all analyses, the following fossil-derived maximum and minimum divergence times were used as calibrations as follows: Dermochelyidae–Cheloniidae 100–150 Ma [1,2], Cheloniidae 50–75 Ma [1,37], *Caretta*–*Lepidochelys* 12–20 Ma [36,38–40] and *Lepidochelys* 4.5–5 Ma [37,38]. All calibrations were defined using log-normal prior distributions, as there was no a priori information on the probability functions for any of the fossil data. A Calibrated Yule speciation model [41] was used as the tree prior, appropriate for modeling divergence between species, while still accounting for within-species divergence.

To infer the ancestral distribution of *D. coriacea* haplotypes across oceanic regions, we assigned three geographic states based on the haplotype occurrence, as follows: (A) Pacific Ocean, (B) Atlantic Ocean, and (C) Indian Ocean. The demarcation between the Pacific and Indian Oceans followed the regional designations reported in the original studies from which sequences were obtained, without imposing a strict biogeographic boundary, particularly in the Indo-Western-Pacific region where such limits are inherently complex. In this context, haplotypes from Southeast Asia and adjacent regions were assigned according to their reported ocean basin, recognizing that this approach may simplify underlying biogeographic structure. Given current sampling limitations in the Indo-Western-Pacific, we did not further subdivide the Pacific into western and eastern regions. While alternative partitioning schemes could influence phylogenetic relationships and ancestral state inferences, especially under scenarios proposing an Indo-Western-Pacific source for post-Pleistocene dispersal, the present approach prioritizes consistency and comparability across datasets. For the orphaned Dc1.7 haplotype, we indicated that its origin could span regions (A, B, and C), since it is based on only one sequence (OQ621749) corresponding to a stranded individual found in a foraging area in Uruguay, whose nesting origin remains unknown. The complete set of trees, along with the consensus phylogenetic tree generated by TreeAnnotator 2.7.7 [42], was used as input, and the analysis was conducted under the S-DIVA model [43] in RASP 4 [44], using default parameters. Chronostratigraphic terminology and age assignments followed [45].

## Results

**Fig 1:**
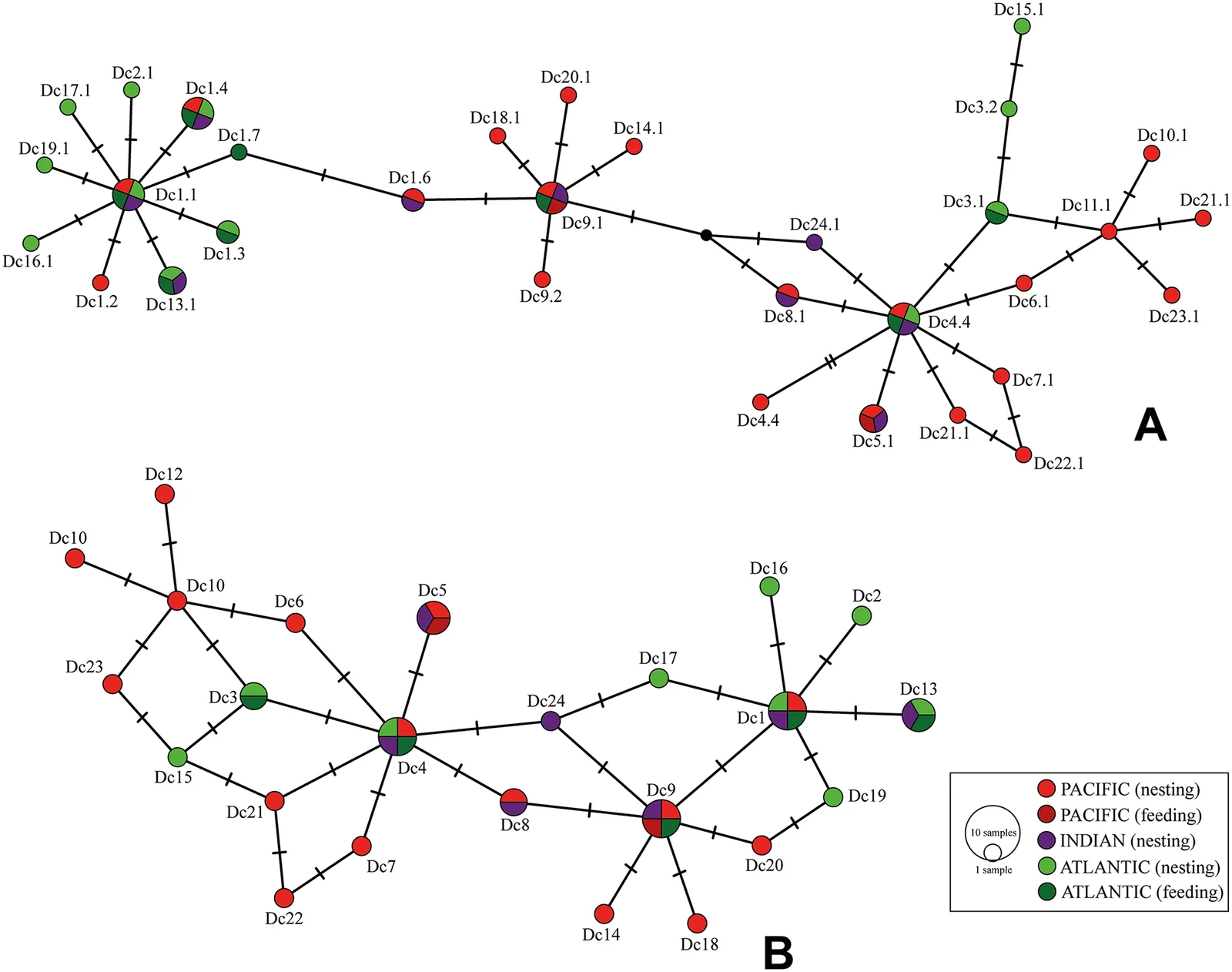
Haplotype network of *D. coriacea* based on mtDNA sequences. **(A)** Long sequence alignment (681 bp) and **(B)** Short sequence alignment (473 bp). Circles represent haplotypes, with their sizes corresponding to the number of oceanic regions where each haplotype was detected, rather than haplotype frequency, since the analysis was based solely on presence/absence data. Connecting lines indicate single-nucleotide differences, with hatch marks denoting multiple mutational steps. Colors represent different oceanic regions where each haplotype was found.

Analysis of 304 sequences revealed 32 haplotypes and 21 variable sites in the long-sequences (681 bp; Table 1; Fig 1A; S2 File), and 24 haplotypes with 14 variable sites in the short-sequences (473 bp; Table 2, Fig 1B; S3 File).

**Table 1:** Polymorphic sites in the mtDNA control region (681 bp) of *D. coriacea* haplotypes. Each row represents a haplotype, with variable sites highlighted at specific base positions. Dots (.) indicate sequence identity with the reference haplotype (Dc1.1). Positions marked with an asterisk (*) and shown in bold represent polymorphic sites reported in previous studies [8], realigned to match the length of our 681 bp sequence. Bases are color-coded to highlight variation: Adenine (A) in green, Thymine (T) in red, Cytosine (C) in blue, and Guanine (G) in purple.

Haplotypes	Variable sites																				
This study	120	185	191	198	199	216	220	229	230	253	278	344	385	400	569	592	602	652	660	664	673
[8]	134*	199*	205	212*	213*	230	234	243*	244	267	292*	358	399	414	583	606	616*	666	674*	678*	687*
Dc1.1	G	A	T	G	C	T	G	G	T	T	A	A	A	T	A	A	A	A	C	T	T
Dc1.2	.	.	.	.	.	.	.	.	.	.	.	.	.	.	G	.	.	.	.	.	.
Dc1.3	.	.	.	.	.	.	.	.	.	.	.	.	.	.	.	.	.	.	.	.	C
Dc1.4	.	.	.	.	.	.	.	.	.	.	.	.	.	.	.	.	.	.	T	.	.
Dc1.6	.	.	.	.	.	.	.	.	.	.	.	.	.	.	.	G	G	.	.	.	.
Dc1.7	.	.	.	.	.	.	.	.	.	.	.	.	.	.	.	G	.	.	.	.	.
Dc2.1	.	.	.	.	T	.	.	.	.	.	.	.	.	.	.	.	.	.	.	.	.
Dc3.1	A	G	.	A	.	.	.	.	.	.	G	.	.	.	.	.	G	.	.	.	.
Dc3.2	A	G	.	A	.	.	.	.	.	.	G	.	.	.	.	.	G	.	.	C	.
Dc4.1	.	G	.	A	.	.	.	.	.	.	G	.	.	.	.	.	G	.	.	.	.
Dc4.4	.	G	.	A	.	.	.	.	.	.	G	.	.	.	.	.	.	.	T	.	.
Dc5.1	.	G	.	A	.	.	.	.	.	.	G	G	.	.	.	.	G	.	.	.	.
Dc6.1	.	G	.	A	.	.	.	.	.	C	G	.	.	.	.	.	G	.	.	.	.
Dc7.1	.	G	.	A	.	.	.	.	C	.	G	.	.	.	.	.	G	.	.	.	.
Dc8.1	.	.	.	A	.	.	.	.	.	.	G	.	.	.	.	.	G	.	.	.	.
Dc9.1	.	.	.	.	.	.	.	.	.	.	G	.	.	.	.	G	G	.	.	.	.
Dc9.2	.	.	.	.	.	.	.	.	.	.	G	.	.	.	.	G	G	G	.	.	.
Dc10.1	A	G	C	A	.	.	.	.	.	C	G	.	.	.	.	.	G	.	.	.	.
Dc11.1	A	G	.	A	.	.	.	.	.	C	G	.	.	.	.	.	G	.	.	.	.
Dc12.1	A	G	.	A	.	.	.	.	.	C	G	.	G	.	.	.	G	.	.	.	.
Dc13.1	.	.	.	.	.	.	.	A	.	.	.	.	.	.	.	.	.	.	.	.	.
Dc14.1	.	.	.	.	.	.	A	.	.	.	G	.	.	.	.	G	G	.	.	.	.
Dc15.1	A	G	.	A	.	G	.	.	.	.	G	.	.	.	.	.	G	.	.	C	.
Dc16.1	.	.	.	.	.	G	.	.	.	.	.	.	.	.	.	.	.	.	.	.	.
Dc17.1	.	G	.	.	.	.	.	.	.	.	.	.	.	.	.	.	.	.	.	.	.
Dc18.1	.	.	.	.	.	.	.	.	.	.	G	.	.	C	.	G	G	.	.	.	.
Dc19.1	A	.	.	.	.	.	.	.	.	.	.	.	.	.	.	.	.	.	.	.	.
Dc20.1	A	.	.	.	.	.	.	.	.	.	G	.	.	.	.	G	G	.	.	.	.
Dc21.1	.	G	.	A	.	G	.	.	.	.	G	.	.	.	.	.	G	.	.	.	.
Dc22.1	.	G	.	A	.	G	.	.	C	.	G	.	.	.	.	.	G	.	.	.	.
Dc23.1	A	G	.	A	.	G	.	.	.	C	G	.	.	.	.	.	G	.	.	.	.
Dc24.1	.	G	.	.	.	.	.	.	.	.	G	.	.	.	.	.	G	.	.	.	.

**Table 2:** Polymorphic sites in the mtDNA control region (473 bp) of *D. coriacea* haplotypes. Each row represents a haplotype, with variable sites highlighted at specific base positions. Dots (.) indicate sequence identity with the reference haplotype (A). Positions marked with an asterisk (*) and shown in bold represent polymorphic sites reported in previous studies [8,9], realigned to match the length of our 473 bp sequence. Bases are color-coded to highlight variation: Adenine (A) in green, Thymine (T) in red, Cytosine (C) in blue, and Guanine (G) in purple.

Haplotypes	Variable sites													
Current study	91	156	162	169	170	187	191	200	201	224	249	315	356	371
[9]	114*	179*	185	192*	193*	210	214	223	224*	247*	272*	338*	379*	394
[8]	134*	199*	205	212*	213*	230	234	243*	244	267	292*	358	399	414
Dc1	G	A	T	G	C	T	G	G	T	T	A	A	A	T
Dc2	.	.	.	.	T	.	.	.	.	.	.	.	.	.
Dc3	A	G	.	A	.	.	.	.	.	.	G	.	.	.
Dc4	.	G	.	A	.	.	.	.	.	.	G	.	.	.
Dc5	.	G	.	A	.	.	.	.	.	.	G	G	.	.
Dc6	.	G	.	A	.	.	.	.	.	C	G	.	.	.
Dc7	.	G	.	A	.	.	.	.	C	.	G	.	.	.
Dc8	.	.	.	A	.	.	.	.	.	.	G	.	.	.
Dc9	.	.	.	.	.	.	.	.	.	.	G	.	.	.
Dc10	A	G	C	A	.	.	.	.	.	C	G	.	.	.
Dc11	A	G	.	A	.	.	.	.	.	C	G	.	.	.
Dc12	A	G	.	A	.	.	.	.	.	C	G	.	G	.
Dc13	.	.	.	.	.	.	.	A	.	.	.	.	.	.
Dc14	.	.	.	.	.	.	A	.	.	.	G	.	.	.
Dc15	A	G	.	A	.	G	.	.	.	.	G	.	.	.
Dc16	.	.	.	.	.	G	.	.	.	.	.	.	.	.
Dc17	.	G	.	.	.	.	.	.	.	.	.	.	.	.
Dc18	.	.	.	.	.	.	.	.	.	.	G	.	.	C
Dc19	A	.	.	.	.	.	.	.	.	.	.	.	.	.
Dc20	A	.	.	.	.	.	.	.	.	.	G	.	.	.
Dc21	.	G	.	A	.	G	.	.	.	.	G	.	.	.
Dc22	.	G	.	A	.	G	.	.	C	.	G	.	.	.
Dc23	A	G	.	A	.	G	.	.	.	C	G	.	.	.
Dc24	.	G	.	.	.	.	.	.	.	.	G	.	.	.

For the long-sequence alignment, Dc1.1 haplotype was the most broadly represented, with 127 sequences (~46.5%), followed by Dc4.1, which was observed in 47 sequences. Some haplotypes showed intermediate representation, such as Dc9.1, which had 19 sequences. Several other haplotypes, including Dc16.1, Dc10.1, Dc12.1, and Dc20.1, were recorded only once in the dataset (Table 3).

**Table 3:** Synonymization of *D. coriacea* haplotypes across different studies. The table presents equivalent haplotype designations from previous studies and their corresponding standardized nomenclature adopted here, for both 681 bp (long) and 473 bp (short) mtDNA sequences. Columns indicate synonymous names from previous publications, while the standardized haplotype names proposed in this study are shown in bold. Newly assigned haplotypes are indicated with an asterisk (*). The TOTAL column represents the number (N) of GenBank sequences matching each standardized haplotype by sequence identity, without reflecting natural population frequencies. The designation “Hap1 (*part*)” refers to a subset of sequences originally grouped under this name, now assigned to distinct standardized haplotypes.

Haplotypes (681 bp)				TOTAL	Haplotypes (473 bp)	TOTAL
**Dc1.1**				127	**Dc1**DcA [9]	199
Dc2 [13]	R [27]	Hap2 [12]	Hap1 (*part*) [12] - OK040246			
**Dc1.2**				1		
**Dc1.3**				3		
Dc3 [13]						
**Dc1.4**				47		
Dc7 [13]		Hap1 (*part*) [12]				
**Dc1.6**				1		
**Dc1.7**				1		
**Dc2.1**				2	**Dc2**DcB [9]	3
**Dc3.1**				16	**Dc3**DcC [9]	27
Dc3 [13]						
**Dc3.2**				9		
**Dc4.1**				3	**Dc4**DcD [9]	5
Dc5 [13]						
Dc4.4*				1		
S [27]						
**Dc5.1**				2	**Dc5**DcE [9]	3
JD3 [22]						
**Dc6.1**				1	**Dc6**DcF [9]	2
**Dc7.1**				1	**Dc7**DcG [9]	2
**Dc8.1**				1	**Dc8**DcH [9]	2
**Dc9.1**				19	**Dc9**DcI [9]	21
Dc6 [13]	JD1 [22]	JD2 [22]				
**Dc9.2**				1		
**Dc10.1**				1	**Dc10**	1
**Dc11.1**				2	**Dc11**DcK [9]	3
Q [27]						
**Dc12.1**				1	**Dc12**DcL [9]	2
**Dc13.1**				12	**Dc13**	12
Dc4 [13]		Hap3 [12]				
**Dc14.1**				1	**Dc14**	1
Dc15.1*				3	**Dc15**	3
C3 [11]						
Dc16.1*				7	**Dc16**	7
A5 [11]						
**Dc17.1**				2	**Dc17**	2
**Dc18.1**				1	**Dc18**	2
**Dc19.1**				2	**Dc19**	2
**Dc20.1**				1	**Dc20**	1
Dc21.1*				1	**Dc21**	1
H2 [14]						
Dc22.1*				1	**Dc22**	1
H3 [14]						
Dc23.1*				1	**Dc23**	1
H4 [14]						
**Dc24.1**				1	**Dc24**	1

In the short-sequence alignment, Dc1 was the most broadly represented haplotype, present in 199 sequences (~65.46%). Dc3 was the second most represented, appearing in 27 sequences, while Dc9 was recorded in 21 sequences. Several haplotypes, including Dc10, Dc14, Dc20, Dc21, Dc22, Dc23, and Dc24, were observed only once in the dataset (Table 3).

For the long-sequence alignment, the haplotype Dc2 was found to be synonymous with R [27], Hap2 [12], and a subset of the sequences previously identified as Hap1 [12], all of which are synonymous with Dc1.1. Likewise, Dc7 [13] corresponds to another subset of Hap1 [12] and is synonymous with Dc1.4, while Dc6 [13] corresponds to JD1 and JD2 [22], and is synonymous with Dc9.1. Additionally, Dc4 [13] and Hap3 [12] were determined to be synonymous with Dc13.1 (Table 3). Several haplotypes were also assigned new standardized names. The haplotype S [27] is now referred to as Dc4.4. Additionally, C3 [11] has been standardized as Dc15.1, A5 [11] as Dc16.1, H2 [14] as Dc21.1, H3 [14] as Dc22.1, and H4 [14] as Dc23.1 (Table 3).

For the short-sequence alignment, 12 new haplotype names were proposed, and the prefix “Dc” was added to the original letter designations of the 11 haplotypes described by [9]. The short-sequence haplotypes Dc10, Dc13, Dc14, Dc15, Dc17, Dc18, Dc19, Dc20, Dc21, Dc22, Dc23, and Dc24 correspond to the long-sequence haplotypes Dc10.1, Dc13.1, Dc14.1, Dc15.1, Dc17.1, Dc18.1, Dc19.1, Dc20.1, Dc21.1, Dc22.1, Dc23.1, and Dc24.1, respectively (Table 3).

When comparing the haplotypes from short sequences with those from long sequences, all six haplotypes related to Dc1 (Dc1.1–Dc1.4, Dc1.6, and Dc1.7) in the long-sequence alignment correspond to the short-sequence haplotype Dc1. Similarly, Dc2.1 in the long-sequence alignment corresponds to Dc2, Dc3.1 and Dc3.2 correspond to Dc3, Dc4.1, and Dc4.4 correspond to Dc4, and Dc9.1 and Dc9.2 correspond to Dc9 (Table 3).

In the long-sequence haplotype network, Dc1.1 haplotype was the most geographically widespread, acting as a central node connected to multiple derived haplotypes through single-nucleotide substitutions (Fig 1A). Dc4.1 and Dc9.1 were also broadly distributed, forming secondary hubs in the network. Several haplotypes showed more restricted distributions, occurring in fewer oceanic regions and occupying peripheral positions in the network as geographically limited variants (Fig 1A). In contrast, Dc1 was the most geographically widespread, like Dc1.1 in the long-sequence network (Fig 1B). Several haplotypes (e.g., Dc3, Dc4 and Dc9) exhibited broader distributions, while numerous haplotypes with more restricted geographic ranges appeared at the periphery (Fig 1B). The general topology remained consistent, with widely distributed haplotypes maintaining similar connectivity patterns as seen in the long-sequence network.

**Fig 2:**
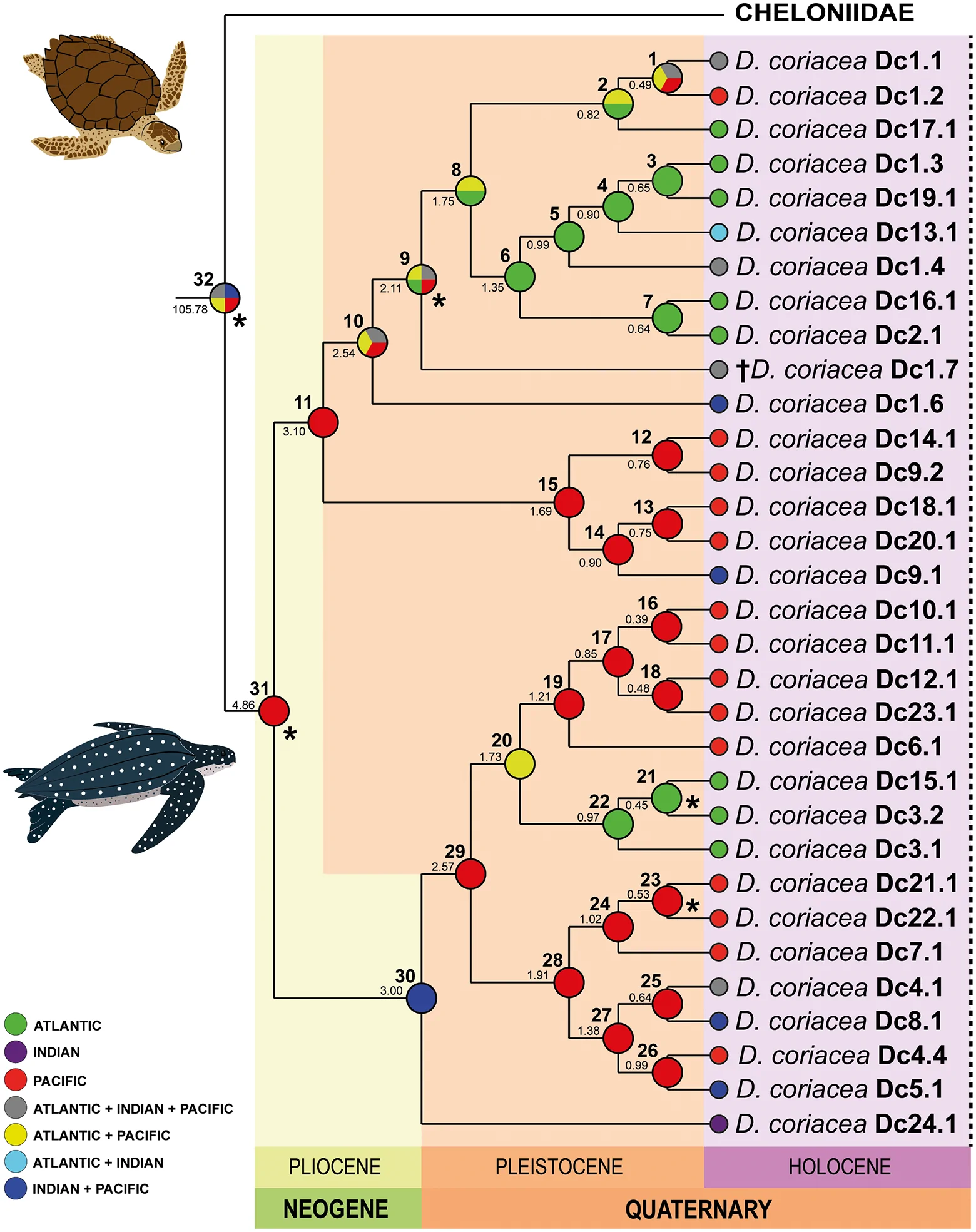
Ancestral area reconstruction for *D. coriacea* haplotypes based on Bayesian phylogeny. Numbers in bold correspond to the main nodes (nodes 1–33). Smaller numerical values adjacent to each node represent the median estimated divergence time in millions of years before present (MYBP). Asterisks (*) indicate nodes with posterior probability support ≥ 0.75. The cross (†) indicates the orphaned haplotype associated with foraging areas. Colored circles adjacent to haplotype names represent their current geographic distributions. The different sizes of the colored sectors within each node indicate the relative probabilities of alternative ancestral area reconstructions inferred for that node.

The ancestral area reconstruction for *D. coriacea* indicated that the most basal node (Fig 2, node 31; S4 Table) within the species was inferred to have a distribution in the Pacific Ocean. From this origin, two major lineages were observed: one leading to a clade composed predominantly of haplotypes found in the Atlantic Ocean (Fig 2, node 11), and another giving rise to haplotypes distributed across the Pacific, Indian, and Atlantic Oceans (Fig 2, node 30).

The lineage containing the most geographically widespread (Fig 2, node 11), such as Dc1.1, Dc1.4, Dc9.1, and Dc13.1, was inferred to have an ancestral distribution in the Pacific Ocean. From this origin, the clade diversified into lineages with ancestral areas reconstructed in the Atlantic Ocean (e.g., Fig 2, nodes 3–7). Subsequently, additional diversification occurred within this lineage, giving rise to haplotypes associated with new ancestral areas in the Pacific and Indian Oceans (e.g., Fig 2, node 10). Some intermediate nodes within this clade also exhibited mixed distributions (Fig 2, nodes 1, 9 and 10), reflecting geographic transitions along the lineage. Meanwhile, one lineage remained in the Pacific Ocean (Fig 2, node 15), maintaining a consistent regional distribution through subsequent diversification events. Notably, the orphaned Dc1.7 haplotype, which previously had an undefined origin, is now more confidently associated with the Atlantic Ocean, based on its position within a cluster of nodes consistently reconstructed with Atlantic ancestry (Fig 2, node 9).

The second major lineage (Fig 2, node 30) comprises a diverse group of *D. coriacea* haplotypes distributed across multiple oceanic regions, with ancestral states predominantly inferred in the Pacific Ocean. From this Pacific-centered lineage, an early vicariance event was inferred at node 30 with full support (probability = 1.0, S5 Appendix), separating lineages associated with the Pacific and Indian oceans. This split resulted in the Pacific lineage (Fig 2, node 29) and to haplotype Dc24.1, which was detected exclusively in the Indian Ocean. Subsequently, within the Pacific lineage, node 20 was reconstructed as occupying both the Pacific and Atlantic oceans. A second vicariance event (probability = 1.0, S5 Appendix) then separated into Pacific (Fig 2, node 19) and Atlantic (Fig 2; node 22) clades. Within the Atlantic lineage, haplotypes Dc3.1, Dc3.2, and Dc15.1 are restricted to the Atlantic Ocean, consistent with diversification following the separation of these oceanic regions.

The divergence time analysis provided a temporal framework for the evolutionary history of *D. coriacea* (Table 4). The most basal split within the Dermochelyidae+Cheloniidae clade was estimated at approximately ~100 million years ago (Ma) (Fig 2, node 32; Table 4), with a 95% highest posterior density (HPD) interval ranging from 117.27 to 100.25 Ma (Table 4). Within *D. coriacea*, the primary divergence separating the two major haplogroup lineages occurred around 4.86 Ma (Fig 2, node 31; Table 4), with a narrower HPD interval between 8.71 and 1.84 Ma (Table 4).

**Table 4:** Calibrations used in molecular clock analyses and posterior TMRCA (time to most recent common ancestor) estimates. The column “Node in Fig 2” refers to the corresponding numbered node in the time-calibrated phylogeny (Fig 2), when applicable. “Prior (MYBP)” indicates the fossil-based calibration interval or tree prior used in the divergence time estimation. “Median (MYBP)” represents the estimated median divergence time in millions of years before present, and “95% HPD” indicates the 95% highest posterior density interval associated with the estimated node age.

Taxonomic group	Node in Fig 2	Prior (MYBP)	Median (MYBP)	95% HPD
Dermochelyidae + Cheloniidae	32	100.00-150.00	105.78	100.25-117.27
Cheloniidae	–	50.00-75.00	53.69	50.37-59.01
Cheloniidae (except for Chelonia + Natator)	–	Tree prior	19.51	13.06-28.07
*Eretmochelys imbricata*	–	Tree prior	4.86	1.01-9.59
Caretta + Lepidochelys	–	12.00-20.00	13.86	12.30-1.04
*Caretta caretta*	–	Tree prior	0.44	0.00-1.47
*Lepidochelys*	–	4.50-5.00	4.81	4.63-5.02
*Lepidochelys kempii*	–	Tree prior	0.95	0.04-2.31
*Lepidochelys olivacea*	–	Tree prior	0.33	0.00-1.08
*Chelonia + Natator*	–	Tree prior	14.20	4.36-26.76
*Chelonia mydas*	–	Tree prior	05.09	1.14-10.27
*Natator depressus*	–	Tree prior	1.34	0.07-3.36
*Dermochelys coriacea (all haplotypes)*	31	Tree prior	4.86	1.84-8.71
*D. coriacea* (nodes 16–29 + Dc24.1)	30	Tree prior	3.00	1.08-5.31
*D. coriacea* (nodes 16–28)	29	Tree prior	2.57	0.91-4.64
*D. coriacea* (nodes 23–27)	28	Tree prior	1.91	0.55-3.56
*D. coriacea* (nodes 25–26)	27	Tree prior	1.38	0.28-2.85
*D. coriacea* (Dc4.4 + Dc5.1)	26	Tree prior	0.99	0.10-2.24
*D. coriacea* (Dc4.1 + Dc8.1)	25	Tree prior	0.64	0.01-1.66
*D. coriacea* (node 23 + Dc7.1)	24	Tree prior	1.02	0.11-2.28
*D. coriacea* (Dc21.1 + Dc22.1)	23	Tree prior	0.53	0.01-1.41
*D. coriacea* (node 21 + Dc3.1)	22	Tree prior	0.97	0.11-2.12
*D. coriacea* (Dc15.1 + Dc3.2)	21	Tree prior	0.45	0.01-1.21
*D. coriacea* (nodes 16–19 + 21–22)	20	Tree prior	1.73	0.52-3.31
*D. coriacea* (nodes 16–18 + Dc6.1)	19	Tree prior	1.21	0.25-2.48
*D. coriacea* (Dc12.1 + Dc23.1)	18	Tree prior	0.48	0.03-1.14
*D. coriacea* (nodes 16 + 18)	17	Tree prior	0.85	0.13-1.80
*D. coriacea* (Dc10.1 + Dc11.1)	16	Tree prior	0.39	0.00-0.99
*D. coriacea* (nodes 12–14)	15	Tree prior	1.69	0.26-3.57
*D. coriacea* (node 13 + Dc9.1)	14	Tree prior	0.90	0.08-2.08
*D. coriacea* (Dc18.1 + Dc20.1)	13	Tree prior	0.75	0.05-1.87
*D. coriacea* (Dc14.1 + Dc9.2)	12	Tree prior	0.76	0.03-1.87
*D. coriacea* (nodes 1–10 + 12–15)	11	Tree prior	3.10	1.04-5.64
*D. coriacea* (nodes 1–9 + Dc1.6)	10	Tree prior	2.54	0.77-5.01
*D. coriacea* (nodes 1–8 + Dc1.7)	9	Tree prior	2.11	0.58-4.03
*D. coriacea* (nodes 1–7)	8	Tree prior	1.75	0.47-3.56
*D. coriacea* (Dc16.1 + Dc2.1)	7	Tree prior	0.64	0.04-1.50
*D. coriacea* (nodes 3–5 + 7)	6	Tree prior	1.35	0.37-2.73
*D. coriacea* (nodes 3–4 + Dc1.4)	5	Tree prior	0.99	0.17-1.89
*D. coriacea* (node 3 + Dc13.1)	4	Tree prior	0.90	0.10-2.06
*D. coriacea* (Dc1.3 + Dc19.1)	3	Tree prior	0.65	0.05-1.55
*D. coriacea* (node 1 + Dc17.1)	2	Tree prior	0.82	0.11-1.81
*D. coriacea* (Dc1.1 + Dc1.2)	1	Tree prior	0.49	0.00-1.25

The lineage that includes the most broadly represented haplotypes (Fig 2, node 11; Table 4) was dated to approximately 3.10 Ma, with an HPD ranging from 5.64 to 1.04 Ma (Table 4). The node directly ancestral to Dc1.1, the most extensively represented haplotype, was estimated at 0.94 Ma (Fig 2, node 1; Table 4), with an HPD interval of 1.25 to 0.00 Ma (Table 4). Two distinct colonization events into the Atlantic Ocean were identified from Pacific-origin lineages. The first event was estimated at approximately 2.54 Ma (Fig 2, node 10; Table 4), with a 95% HPD interval between 5.02 to 0.77 Ma (Table 4). The second event was slightly more recent, with an estimated divergence time of 1.21 Ma (Fig 2, node 19; Table 4) and an HPD interval of 2.48 to 0.25 Ma (Table 4).

Across the phylogeny, more recent nodes generally exhibited narrower HPD intervals (see Table 4), while deeper splits displayed wider error margins, consistent with expected differences in temporal resolution due to the placement of fossil calibration points and substitution rate variation across lineages.

## Discussion

The standardization of haplotype nomenclature introduced in this study is a key contribution to the field of sea turtle phylogeography. The nomenclature inconsistency across studies has long been a limiting factor in comparative analyses, making it difficult to integrate datasets from different regions and research groups. For instance, as longer sequences (e.g., 763 bp) [8] began to be more commonly used alongside shorter sequences (e.g., 496 bp) [9], it became necessary to establish an organized and standardized system to avoid redundancy and minimize the risk of misinterpretations in population assessments. By establishing a standardized system for both short (473 bp) and long sequences (681 bp), we ensure that future research can compare datasets without ambiguity, leading to more precise assessments of genetic diversity, connectivity, and evolutionary patterns.

Our findings highlight the importance of reevaluating previous studies that relied on inconsistent haplotype nomenclature, as the lack of standardization may have led to overestimation or underestimation of genetic diversity. For example, our reclassification shows that haplotypes previously considered distinct, such as Dc7 [13] and Hap1 [12], are, in fact, synonymous with Dc1.4. Similarly, Dc2 [13] is equivalent to Hap2 [12] and Dc1.1, which has implications for the interpretation of connectivity patterns between South Atlantic and Indo-Pacific populations. If previous studies had overestimated the number of unique haplotypes, this could have influenced conclusions regarding the level of genetic differentiation between nesting colonies, leading to misinformed conservation priorities.

Furthermore, our study provides additional context for interpreting the relationship between haplotypes detected in foraging areas and nesting populations. Rather than directly inferring connectivity, as in mixed-stock analyses, our phylogenetic framework complements these approaches by incorporating orphan haplotypes and placing them within a global evolutionary context. For example, the phylogenetic placement of the orphan haplotype Dc1.7 within an Atlantic-associated lineage helps constrain its likely origin and rule out distant ocean basin sources. This contribution complements previous studies demonstrating that the Southwest Atlantic foraging grounds host individuals from diverse natal origins, particularly West Africa [19], by refining hypotheses about the origin of individuals lacking known nesting provenance. These findings reinforce the importance of integrating phylogenetic and population-level approaches to better interpret the origin of individuals in foraging areas, without directly inferring connectivity patterns [12,18].

The genetic connectivity between Pacific, Indian, and Atlantic subpopulations reflects the broad dispersal and migratory capacity of *D. coriacea*, but also highlights remaining gaps in our understanding of genetic diversity and population structure across oceanic regions [45]. Earlier studies reported two highly divergent haplotypes from Sumatra, Dc4.2 and Dc4.3, which showed unusually long branch lengths compared to other haplotypes. These sequences were initially interpreted as potentially representing deeply divergent lineages or other sources of genetic variation. However, subsequent analyses demonstrated that these haplotypes could not be validated and were most likely the result of sequencing errors. Dc4.2 and Dc4.3 could not be validated in additional samples from Sumatra and are now considered to result from sequencing errors; therefore, these haplotypes should not be included in future analyses [17]. Accordingly, they were excluded from the present study.

Significant genetic differentiation among nesting colonies at varying spatial and temporal scales corroborates earlier findings and reinforces the existence of clearly defined management units critical for targeted conservation policies [7,8,11]. In Asian regions, particularly in Japanese waters, the dominance of haplotypes common to Western Pacific populations highlights strong natal site fidelity and defined migratory routes, directly influencing regional conservation and management strategies [22].

Standardizing haplotype classification enhances the clarity of genetic connectivity patterns, enabling more reliable conservation assessments and facilitating collaboration across research groups. Recently, an open-access sea turtle mtDNA database was established to provide a resource for standardizing haplotype nomenclature and facilitating data sharing across studies [46]. Information from our study provides a verified leatherback dataset that compiles all haplotypes published to date, creating a robust baseline that can be dynamically updated as new data become available, thereby further enhancing standardization and collaboration among research groups. Genetic differentiation among nesting colonies is an important factor in defining RMUs and broader regional management frameworks [3,5]. By providing a more cohesive system, our standardized nomenclature allows researchers, conservation practitioners, and policymakers to interpret genetic data better, facilitating the identification of key nesting and foraging sites and enhancing efforts to mitigate threats such as bycatch, habitat loss, and climate change.

Moreover, the identification of synonymous haplotypes across regions underscores the need for multinationalcollaboration in conservation policies. For example, if Brazilian and West African populations share common haplotypes, conservation efforts in one region will directly impact the viability of populations in another. Rather than directly informing short-term management actions, the primary contribution of this study lies in providing a standardized global framework for interpreting haplotype diversity across ocean basins. This is particularly relevant for long-term conservation planning, as it enables more accurate comparisons across studies, improves the reconstruction of historical connectivity, and establishes a robust baseline for detecting future changes in distribution patterns, especially under scenarios of climate-driven shifts in ocean circulation and habitat use.

The ancestral area reconstruction and divergence time estimates presented in this study provided important insights into the evolutionary history and global dispersal of *D. coriacea*. Our analysis identified the Pacific Ocean as the most likely ancestral distribution for the species (Fig 2, node 31), consistent with previous studies suggesting an Indo-Pacific origin for sea turtle lineages before subsequent colonization of the Atlantic Ocean [9,27]. We estimated the MRCA of all *D. coriacea* haplotypes at approximately 4.86 Ma, during the late Miocene to early Pliocene (Fig 2, node 31; Table 4). This estimate reflects the crown age of extant mitochondrial lineages. In contrast, a previous study [27] estimated a much more recent mitochondrial divergence time for *D. coriacea*, at approximately 0.17 Ma (95% HPD: 0.06–0.35 Ma), based on only three haplotypes (corresponding to Dc1.1, Dc11.1, and Dc16.1). The older divergence time recovered here is likely explained by the inclusion of a substantially larger dataset comprising 32 haplotypes, capturing a broader representation of the known mitochondrial diversity of *D. coriacea*, which improves the resolution of divergence time estimates compared to analyses based on limited haplotype sampling. Fossil evidence indicates that dermochelyids were highly diverse during the early Tertiary, with multiple species recognized across various regions [47]. However, by the end of the Miocene, this diversity had markedly declined, and only *D. coriacea* persisted into the Pliocene and continues to the present day [47]. Thus, although our molecular estimate of ~4.86 Ma likely captures the timing of diversification among surviving mtDNA lineages, the true origin of *D. coriacea* as a species may predate this estimate.

The divergence of major *D. coriacea* lineages, including the Atlantic clade, likely occurred during and after the closure of the Isthmus of Panama, estimated at ~3.0 million years ago (Ma). This key vicariant event disrupted gene flow between the Pacific and Atlantic Oceans and profoundly shaped marine biogeography [9,48,49]. Our results support this scenario, with two distinct colonization events from the Pacific into the Atlantic inferred at approximately ~1.35–0.97 Ma and ~0.64 Ma (Fig 2, nodes 6, 7, and 22), during the early Pleistocene. These events may reflect different dispersal mechanisms across time.

The first colonization (~3.10–2.54 Ma; Fig 2, nodes 10 and 11) likely coincided with the final stages of the closure of the Isthmus of Panama. This geological transformation restructured global ocean circulation, severing direct marine connections between the Pacific and Atlantic, and possibly constraining gene flow. One potential southern route involves the Agulhas Leakage, a system of warm-water eddies that sporadically transports Indian Ocean waters into the South Atlantic [50]. The second colonization (~0.97 Ma, Fig 2, node 22) may have been associated with intensified Pleistocene glacial–interglacial cycles, which periodically reshaped equatorial current systems and thermal gradients, reducing biogeographic barriers across the Atlantic [51]. These conditions may have enabled east-to-west dispersal through the equatorial Atlantic, particularly during interglacial periods when ocean temperatures and current velocities favored long-distance movements by pelagic species [52–54]. Before these events, the ancient Tethys Sea, which began closing during the Eocene (~40 Ma) and was fully closed by the early Pliocene (~5.0–4.0 Ma), served as a vital marine corridor between the Indo-Pacific and the proto-Mediterranean-Atlantic regions [55]. Although it was likely not directly used by *D. coriacea*, its closure played a foundational role in shaping the oceanic connectivity that followed, influencing subsequent routes of dispersal and colonization.

The median divergence time estimated for all *D. coriacea* haplotypes was ~ 0.97 Ma, aligning with the species’ relatively shallow mitochondrial divergence [27], consistent with recent colonization and extensive transoceanic movements that mitigate genetic structure [27,52]. In contrast, other species such as *E. imbricata* [53] or *C. mydas* [54], show earlier divergence and deeper population structure.

Collectively, these findings support a dynamic evolutionary history for *D. coriacea*, shaped by a combination of dispersal, colonization, and climatic shifts over the Pleistocene. Understanding this evolutionary context is essential for interpreting current patterns of genetic diversity and for informing transoceanic conservation strategies. Our results highlight the importance of using longer sequence data whenever possible, as shorter sequences may fail to capture finer-scale population structure. While short sequences remain valuable, particularly in studies involving stranded animals where sample degradation can compromise DNA quality and limit sequence length, longer sequences provide greater resolution and should be prioritized whenever feasible. This recommendation aligns with recent studies [12,22] demonstrating how extended sequences can reveal previously undetected diversity. Future research should prioritize whole-mitochondrial genome sequencing and complementary nuclear markers to further refine our understanding of population connectivity.

In conclusion, the haplotype nomenclature standardization adopted in this study represents a critical advancement for integrating genetic data and refining population connectivity assessments in *D. coriacea*. By establishing a unified system, we overcome a major barrier in sea turtle phylogeography, enabling more effective conservation planning. Future research should build upon this framework with genomic approaches, expanded regional sampling, and further investigation into the influence of NUMTs.

## Supporting information

S1 Table Haplotype frequencies of *D. coriacea* based on the mtDNA control region. (XLSX)

S2 File Leatherback turtle (*D. coriacea*) mtDNA control region long sequences (681 bp). (CSV)

S3 File Leatherback turtle (*D. coriacea*) mtDNA control region short sequences (473 bp). (CSV)

S4 Table Biogeographic events inferred from the S-DIVA analysis in RASP for *D. coriacea.* For each node, the number of dispersal, vicariance, and extinction events, the inferred event route, and the associated probability are reported. Ocean codes: A = Pacific, B = Atlantic, C = Indian.
(XLSX)
