# Supplementary material for: Haplotypes across the Oceans: worldwide phylogeography, evolution, conservation and nomenclature standardization in the leatherback sea turtle (Dermochelys coriacea)
Source: PLoS One. 2026 Aug 19;21(8):e0354151. doi: 10.1371/journal.pone.0354151 (PMC13489440; doi:10.1371/journal.pone.0354151)
Supplement: Supplementary file 1 [file pone.0354151.s005.docx]

Review of PONE-D-25-32257: Colombo et al. “Haplotypes across the Oceans: worldwide phylogeography, evolution, conservation and nomenclature standardization in the leatherback sea turtle (*Dermochelys coriacea*)”

By Peter Dutton (Peter.Dutton@noaa.gov)

General comments:

This manuscript addresses an ongoing problem of confusing nomenclature for leatherback turtle Control Region mtDNA haplotypes in the literature, a primary genetic marker for sea turtle population genetic studies. While standardized nomenclature has been in use as proposed by Dutton et al 2013, it’s effective management (e.g. designating names to new haplotypes as they are discovered in a systematic way) and has relied on informal working group networking, which has not been adequate as more researchers and students conduct mtDNA sequencing studies. The problem is further compounded by use of longer sequences that increasingly characterize more recent studies compared with older historic studies based on shorter sequences. With this manuscript, the authors make a very important contribution, by consolidating and analyzing all the previously published sequence data in order to cross reference equivalent haplotypes into a standardized nomenclature. They further conduct a global phylogenetic analysis with this standardized dataset. This is a tremendously useful resource, and overall, a well written paper, however there are some major problems that need to be addressed before this can be published (in its current state, it risks adding further confusion, which undermines the goal of their efforts).

Major issues:

The authors have

1) missed an opportunity to critically evaluate at least one study that introduces significant errors, and thus by including Dc4.2 and Dc4.3 (Maslim et al. 2016) undermines the validity of the network and phylogenetic analysis. See below for details.
2) incorrectly incorporated an Atlantic dataset into the Pacific dataset, further undermining the validity of the phylogeographic analyses.
3) failed to incorporate in progress and new haplotypes that were not available on GenBank at the time their analysis was done, thus leading to duplication of “new” haplotype designations. This is understandable and justifiable, but will lead to even more confusion if not remedied. I have provided details and suggestions below, with the intent of encouraging a more up-to-date paper that could be a tremendously useful resource.
4) The proposed alphabetic nomenclature for the shorter sequences is problematic and counter to a more generally adopted approach adopted for other sea turtle species, including recently for leatherbacks (Toha et al. 2025).
5) The authors could broaden the Introduction to frame the topic of inconsistent nomenclature to other species of sea turtles by including other papers that are relevant, particularly
a. Jensen et al. 2019. The evolutionary history and global phylogeography of the green turtle (*Chelonia mydas*). J. Biogeogr., 46(5), pp.860-870. doi.org/10.1111/jbi.13483
b. Shamblin et al 2017. JEMBE 488:111-120 <https://doi.org/10.1016/j.jembe.2016.11.009> (green turtles, uses haplotype nomenclature that reconcile shorter and longer sequence data)
c. Shamblin et al. 2014. PLoS ONE 9(1): e85956. doi:10.1371/ journal.pone.0085956 (Loggerhead turtles). This paper incorporates shorter and longer mtDNA sequence data as part of a Woking Group to coordinate nomenclature among different research groups.

There are other examples, but these are particularly relevant.

Below, I detail out comments, concerns and suggestions, including minor suggested edits.

Abstract

Line 27 replace “known” with “published” or “publicly available”

Line 57: from this point on it is preferable to refer to “leatherback turtles” rather than “leatherback sea turtles”

Lines 71-72: Toha et al. (2025) moved away from the older alphabetical haplotype nomenclature to adopt more informative nomenclature that reconciles the longer sequence haplotypes with corresponding shorter haplotypes (in order to incorporate older published shorter sequence datasets). This should be stated here in the Introduction, and form the basis for the new nomenclature for the shorter haplotypes.

Methods

Line 133-134: I would highly recommend using the numerical system for the shorter haplotype nomenclature. There are several reasons for doing this, primarily to integrate longer and shorter haplotype names in a more intuitive way that reflects equivalent variants (see comment above re. Toha et al. 2025). Also, it does not limit the number of haplotypes to 26. To date there are a total of 28 unique haplotypes, including the ones identified in this manuscript, and likely to be more into the future.

Line 135 – Provide more detail on how new names/numbers were chosen. The current designations established by Dutton et al. (2013) and in subsequent designations follow a sequential numbering, but there are some “gaps” that are not yet publicly available on GenBank (e.g. Dc15.1, Dc16.1, Dc21.1, etc…see below). While I appreciate that the authors would not have been aware of unpublished haplotypes assigned as part of informal working group haplotype “clearing houses”, it is important to harmonize the new name designations in this paper, with those currently being used by others with manuscripts in review and being submitted on GenBank, to avoid adding further confusion. I am happy to provide additional GenBank sequences for the authors to incorporate into this paper.

Line 190-191. Elaborate on what was the demarcation between the Pacific and Indian Oceans, given the biogeographic complexity related to leatherback nesting sites in the Asia-Pacific and Indo-Pacific region, and the hypothesis (by Dutton et al. 1999 and others) that the Indo-Western-Pacific may have been the source of post-Pleistocene radiation/recolonization to the eastern Pacific. Given the spotty data for the Indo-Western-Pacific, perhaps it doesn’t really matter if the West Pacific and East Pacific are lumped together, but it would be good to clarify and consider how, if at all, parsing out these broad regions, might influence results of the phylogenic analyses and inference of ancestral states. Perhaps food for thought in the Discussion?

Also note that the geographic location designations for some Pacific and Atlantic datasets are incorrect (see comment about Table S1 for details). This calls into question the validity of the phylogeographic analyses.

Results

Line 202: All but one of the transversions disappear once the erroneous Dc4.2 and 4.3 haplotypes are removed. The only transversion is T-G introduced by the newly assigned haplotypes at position 216 in this study (Table 1). Table 1 also indicates a transversion for Dc20.1 at this position (which is position 230 in the Dutton et al. 2013 Dc1.1 alignment). However, the original published Dc20.1 Toha et al. 2025 did not report this variable site, so something seems to be amiss. I have not had time to double check the sequences for the original CmP20.1, but the T-G transversion here should be confirmed by the authors to rule out alignment errors or typos in the table.

Lines 233-236: These new assigned haplotype names duplicate others that have been informally assigned as part of unpublished work-in-progress (see comments above in methods). See below for information on each of the specific haplotypes, and suggestions for harmonizing the haplotype names:

Note that Dc1.5 was informally assigned, but subsequently determined to be an error, therefore we skipped that number.

Dc4.2 and Dc4.3 are not valid haplotypes (Maslim et al. 2016) and should be removed, and highlight here for the record that they should not be used. These names were assigned arbitrarily by the author before he was aware of basic best practices for designating new haplotype names. Cite Toha et al (2025) where this point is made (per pers. Comm from the original author, Maslim). Maslim has since redone his study, and completed a comprehensive analysis of these and other Sumatra nesting sites, and confirmed that these haplotypes do not exist (As-singkily et al. in review). Removal of these two sequences will eliminate many of the new variable sites identified in Tables 1 & 2, and errors introduced in the phylogenic analysis.

Dc16.1 – this appears to be a newly assigned haplotype from a sequence extracted from the whole mitogenome published by Duchene et al (2012). It is unclear why it was designated this number. Dc16.1 has been informally assigned to a sequence from a stranded leatherback in the NE Atlantic (e.g. an orphan haplotype) (LaCasella et al. unpublished). I am happy to provide the sequence and release the GenBank ID if the authors are willing to revise their Dc numbering assignments to the new haplotypes they have identified. Note that the sequence the authors propose to assign to Dc16.1 (681bp), would be more appropriately assigned as a variant of Dc4. Since Dc4.2 and 4.3 are not valid haplotypes, but in the literature, I would suggest assigning it Dc4.4 to avoid future confusion. See comment below regarding shorter 473bp haplotype names that should be harmonized to the base Dc1 -aligned names. In this case the proposed Dc4.4 (681bp) would be the equivalent Dc4 (473bp), as established in Toha et al. (2025).

Similarily, Dc15.1 was informally assigned, but not published, since it represents another rare orphan haplotype found in a handful of NE Atlantic foraging and stranded turtles. Again, I’m happy to provide the sequence and Genbank ID for the authors to use in this paper.

Dc21.1-24.1 are new assignments (of haplotypes originally published under different nomenclature) in this manuscript. These have all been informally assigned to current unpublished haplotypes. If the authors are willing to coordinate, I would be happy to provide the sequences to haplotypes we have assigned as Dc21.1, Dc22.1, Dc23.1 and Dc24.1, to include them in the list. Ideally, the authors would be willing to rename their new haplotypes Dc25.1-Dc28.1, and insert the ones we have already designated, since two of the haplotypes are part of manuscripts currently in review. Our working list only goes through Dc24.1, so this paper would be a timely extension of all the current work in progress.

Table caption (line 250-261). The names in bold represent both previously assigned and the newly assigned haplotypes. This is confusing, please distinguish the current published and assigned **Dc*x.n*** haplotyes from the ones being assigned new names.

Lines 279-281: This is a good point, since we would not expect this stranded turtle to have originated from the Pacific, thus demonstrating the utility of the phylogenetic analysis in this case.

Supplementary Table S1: Some of the locations are incorrect. ACR in Dutton et al. 2013 refers to Atlantic Costa Rica (Gandoca and Tortuguero), but it is listed under the Pacific locations and combined with the sample from Ostional (Pacific) from Duchene et al. 2012 in both the longer and shorter sequence tables. The shorter sequence table has ACR (Tortuguero and Gandoca) correctly under the Atlantic for the data from Dutton et al. 1999, but incorrectly for the Dutton et al. 2013. This brings into question the accuracy of the geographic assignments for the phylogentic analyses, especially if Caribbean data was conflated with eastern Pacific.

Discussion

The Discussion should be revised depending on the outcome of removal of Dc4.2 and 4.3 and correction of the geographic location errors noted above, particularly the paragraphs from lines 438-452.

Line 358: “…this also highlights significant knowledge gaps regarding genetic diversity and population structure within these oceanic regions…” This is true, work in progress is uncovering new diversity (e.g. As-singkily et al. in review), but not at the deep level stated in lines 359-362.

Line 370: Pointing out potential NUMTs is good, there is a need to expand awareness. I’m not sure it explains the errors in the case of the Dc4.2 and Dc4.3 study though. Can the sequence be checked against the known NUMT? Unless you can show these are NUMTs, I’d suggest re-organizing this section to include a paragraph addressing caveats of using poor quality data and also point out the sequences that you ended up discarding (mentioned in methods) because of data quality issues. That would allow you highlight some best practices that you use, and include screening for NUMTs as one additional QA/QC step.

Otherwise, there are some excellent general points presented in thoughtful ways. Some points however could benefit from more specificity.

Line 381- points out the value of establishing a more cohesive system with standardized nomenclature. This would be a good place to mention examples with other species (e.g. the Jensen et al 2018 green turtle and Jensen et al. 2025, Shamblin et al 2017)…that illustrate nomenclatures that reflect intuitive internal cohesion between shorter and longer haplotypes by numbering variants of base shorter haplotypes with a longer sequence variant extension (e.g for CmP4 shorter-CmP4.1, 4.2, 4.3, or for CmA5 shorter-CmA5.1, CmA5.2, etc), and reinforce the preference to use a similar approach for leatherbacks. Again, the use of alphabetical haplotypes for shorter sequences does not offer the same level of cohesion.

Line 350: “our study clarifies the relationship between foraging areas and nesting populations”- it is not clear how the analysis in this manuscript does this…be more specific if possible. Also, a related point in Lines 385-389: this seems like a general vague statement that repeats common statements from other more targeted MSA studies that have already established the connectivity between Brazil (and other foraging grounds in Uruguay and Argentina and nesting populations in West Africa). What additional contribution does this study make? The presence of shared common haplotypes by itself doesn’t necessarily indicate connectivity, but perhaps mentioning that to supplement these MSA studies, the more comprehensive phylogenetic analysis that includes orphan haplotypes can at least help rule out some distant ocean basin origin, as your findings for Dc1.7 illustrate.

Similarly line 388-389, “This highlights the need for coordinated protection measures, particularly in reducing bycatch, and pollution in key migratory corridors” is an obvious often-repeated general statement, but are there specific actions that can be given? While MSA and regional connectivity studies address relatively shorter-term management timescales, identifying synonymous haplotypes on a broad global scale seems be the more relevant application from this study, with management implications on deeper evolutionary time scales (climate change, etc). You could discuss what, if any, the implications are of your global analysis results.
