# Supplementary material for: Haplotypes across the Oceans: worldwide phylogeography, evolution, conservation and nomenclature standardization in the leatherback sea turtle (Dermochelys coriacea)
Source: PLoS One. 2026 Aug 19;21(8):e0354151. doi: 10.1371/journal.pone.0354151 (PMC13489440; doi:10.1371/journal.pone.0354151)
Supplement: Supplementary file 2 [file pone.0354151.s007.docx]

**Dear Dr. Ulrich Joger,**

**We hope this message finds you well. Thank you for your insightful feedback on our manuscript, “*Haplotypes across the Oceans: worldwide phylogeography, evolution, conservation and nomenclature standardization in the leatherback sea turtle (Dermochelys coriacea)*”. We are pleased to submit the revised version of the manuscript, in which we carefully considered and addressed all the reviewers’ suggestions, including yours, as detailed below. As requested, we have uploaded a clean version of the revised manuscript without tracked changes, as well as a marked version highlighting all modifications.**

**Editor Comments:**

The reviewer made very useful suggestions which you should follow. In particular, the fact that haplotypes 4.2 and 4.3 are erroneous, invalidates part of your study. All calculations involving these haplotypes must be redone. In addition, statements like: “unique haplotypes in regions like Sumatra, suggests considerable dispersal and migratory potential. However, this also highlights significant knowledge gaps regarding genetic diversity and population structure within these oceanic regions [17]. Notably, Sumatra haplotypes (Dc4.2 ≈ DcJ, Dc4.3 ≈ DcM) show an exceptionally high level of polymorphism and unusually long branch lengths compared to all other haplotypes analyzed in this study (discussion) must be changed.
**R:** We thank the Editor for this important observation and fully agree. Following this recommendation, we removed haplotypes 4.2 and 4.3 from the dataset because they were identified as sequencing errors by the recently published paper, and we revised the manuscript accordingly. This exclusion is now explicitly stated in the Material and Methods section. All analyses affected by these erroneous haplotypes were redone using the corrected dataset, including haplotype alignments, haplotype counts, variable-site assessments, haplotype networks, synonymization tables, and the subsequent phylogeographic interpretation. The revised version now reports 32 haplotypes in the long-sequence dataset and 24 haplotypes in the short-sequence dataset after these corrections. In addition, we removed the discussion statements that interpreted the former Sumatra haplotypes as highly divergent or unusually polymorphic, since those inferences were based on erroneous sequences and are therefore not valid. The revised manuscript no longer uses haplotypes 4.2 and 4.3 to support claims about exceptional polymorphism, long branch lengths, or dispersal potential associated with Sumatra. The remaining discussion was adjusted to reflect only the validated haplotypes and the corrected analyses.

**Reviewer 1:** *Peter Dutton*

**General comments:**

This manuscript addresses an ongoing problem of confusing nomenclature for leatherback turtle Control Region mtDNA haplotypes in the literature, a primary genetic marker for sea turtle population genetic studies. While standardized nomenclature has been in use as proposed by Dutton et al 2013, it’s effective management (e.g. designating names to new haplotypes as they are discovered in a systematic way) and has relied on informal working group networking, which has not been adequate as more researchers and students conduct mtDNA sequencing studies. The problem is further compounded by use of longer sequences that increasingly characterize more recent studies compared with older historic studies based on shorter sequences. With this manuscript, the authors make a very important contribution, by consolidating and analyzing all the previously published sequence data in order to cross reference equivalent haplotypes into a standardized nomenclature. They further conduct a global phylogenetic analysis with this standardized dataset. This is a tremendously useful resource, and overall, a well written paper, however there are some major problems that need to be addressed before this can be published (in its current state, it risks adding further confusion, which undermines the goal of their efforts).

**R:** We sincerely thank the reviewer for this thoughtful and highly relevant assessment of our manuscript. We greatly appreciate the recognition of the importance and potential utility of our work in addressing the long-standing issue of inconsistent haplotype nomenclature in leatherback turtle mtDNA studies. We fully acknowledge the reviewer’s concern that, in its previous version, the manuscript could inadvertently contribute to further confusion. In response, we have carefully revised the manuscript to ensure clarity, consistency, and robustness of the proposed nomenclature framework and associated analyses.

Specifically, we have:

- Corrected critical data issues, including the removal of erroneous haplotypes (e.g., Dc4.2 and Dc4.3), and recomputed all affected analyses, ensuring that the standardized dataset is accurate and reliable.
- Revised the haplotype cross-referencing system, improving transparency in how previously published haplotypes are matched and synonymized across studies.
- Clarified the criteria used for haplotype designation, particularly in relation to differences between short and long sequence datasets, to avoid ambiguity in future applications.
- Improved the presentation of results and supporting tables, making it easier for readers to trace equivalences among haplotypes and understand how the standardized nomenclature is applied.
- Revised the discussion throughout the manuscript to remove unsupported interpretations and ensure that all conclusions are strictly based on validated data.

We believe these revisions substantially reduce the risk of misinterpretation and significantly strengthen the manuscript’s contribution as a reference framework for future studies. We are grateful for the reviewer’s insights, which have helped us improve both the rigor and clarity of the work.

Major issues:

The authors have

1) missed an opportunity to critically evaluate at least one study that introduces significant errors, and thus by including Dc4.2 and Dc4.3 (Maslim et al. 2016) undermines the validity of the network and phylogenetic analysis. See below for details. **R:** We thank the reviewer for this important comment and fully agree that the inclusion of Dc4.2 and Dc4.3, as reported by Maslim et al. (2016), compromised the interpretation of part of our original analyses. In the revised manuscript, we addressed this issue explicitly and critically. First, we removed Dc4.2 and Dc4.3 from the dataset after recognizing that these haplotypes were erroneous and should not be retained in the standardized nomenclature framework. As a consequence, all analyses affected by their inclusion were redone using the corrected dataset, including the haplotype network, phylogenetic reconstruction, and all related summary calculations. Second, we revised the text to make clear that the previous interpretation associated with these haplotypes was invalid. We removed statements suggesting exceptional divergence, long branch lengths, or unusual polymorphism associated with the Sumatra sequences, since these inferences were based on erroneous haplotypes and were therefore unsupported. Third, we agree that this was an important opportunity to more critically assess problematic records in the literature. Accordingly, the revised manuscript now adopts a more cautious and critical approach when consolidating published haplotypes, emphasizing that literature-derived sequence records must be carefully validated before being incorporated into standardized reference frameworks. We appreciate the reviewer for highlighting this issue, which has significantly improved the rigor, accuracy, and reliability of the revised manuscript.

2) incorrectly incorporated an Atlantic dataset into the Pacific dataset, further undermining the validity of the phylogeographic analyses. **R:** We thank the reviewer for this important clarification and fully agree with this assessment. In the revised manuscript, we have explicitly addressed these issues.

3)	failed to incorporate in progress and new haplotypes that were not available on GenBank at the time their analysis was done, thus leading to duplication of “new” haplotype designations. This is understandable and justifiable, but will lead to even more confusion if not remedied. I have provided details and suggestions below, with the intent of encouraging a more up-to-date paper that could be a tremendously useful resource. **R:** We thank the reviewer for this important and constructive comment. We agree that the dynamic nature of haplotype discovery represents a major challenge for maintaining a consistent and up-to-date nomenclature framework. At the time our analyses were originally conducted, all available sequence data deposited in GenBank and accessible through the literature were included. However, as correctly noted by the reviewer, it is inherently difficult to account for ongoing or unpublished studies at the time of data compilation, which may lead to overlapping or duplicated haplotype designations. Importantly, between the previous version of the manuscript and this resubmission, additional haplotypes have been formally published, and previously included erroneous haplotypes (e.g., Dc4.2 and Dc4.3) have been removed from our dataset. In response, we have updated the dataset and revised the nomenclature framework accordingly. We reorganized the haplotype designation system to incorporate these newly published haplotypes and to avoid duplication or ambiguity in naming. The revised manuscript now reflects an updated and consolidated nomenclature that accounts for both the corrected dataset and the most recent available information. We fully agree with the reviewer that maintaining consistency in haplotype naming requires continuous updates, and we emphasize in the revised manuscript the need for coordinated efforts and centralized curation to minimize future inconsistencies.

4) The proposed alphabetic nomenclature for the shorter sequences is problematic and counter to a more generally adopted approach adopted for other sea turtle species, including recently for leatherbacks (Toha et al. 2025). **R:** We thank the reviewer for this important point and agree that consistency with existing nomenclature systems is essential. In the revised manuscript, we replaced the alphabetic nomenclature for shorter sequences with the numerical system proposed by Toha et al. (2025), ensuring alignment with current standards and improving compatibility across studies.

5) The authors could broaden the Introduction to frame the topic of inconsistent nomenclature to other species of sea turtles by including other papers that are relevant, particularly:

a) Jensen et al. 2019. The evolutionary history and global phylogeography of the green turtle (*Chelonia mydas*). J. Biogeogr., 46(5), pp.860-870. [doi.org/10.1111/jbi.13483](http://doi.org/10.1111/jbi.13483)
b) Shamblin et al 2017. Mexican origins for the Texas green turtle foraging aggregation: A cautionary tale of incomplete baselines and poor marker resolution. JEMBE 488: 111-120. <https://doi.org/10.1016/j.jembe.2016.11.009> [green turtles, uses haplotype nomenclature that reconciles shorter and longer sequence data].
c) Shamblin et al. 2014. PLoS ONE 9(1): e85956. doi:10.1371/ journal.pone.0085956 (Loggerhead turtles). [This paper incorporates shorter and longer mtDNA sequence data as part of a Working Group to coordinate nomenclature among different research groups].

There are other examples, but these are particularly relevant. Below, I detail out comments, concerns and suggestions, including minor suggested edits.

**R:** We thank the reviewer for this valuable suggestion. The recommended references (Jensen et al., 2019; Shamblin et al., 2017; Shamblin et al., 2014) have been incorporated into the Introduction to broaden the context and highlight that challenges related to inconsistent haplotype nomenclature and sequence length integration are not unique to leatherback turtles, but are also recognized in other sea turtle species.

**Abstract**

- **Line 27:** replace “known” with “published” or “publicly available”. **R:** Done.
- **Line 57:** from this point on it is preferable to refer to “leatherback turtles” rather than “leatherback sea turtles”. **R:** Done. We have replaced “leatherback sea turtles” with “leatherback turtles” throughout the manuscript.
- **Lines 71-72:** Toha et al. (2025) moved away from the older alphabetical haplotype nomenclature to adopt more informative nomenclature that reconciles the longer sequence haplotypes with corresponding shorter haplotypes (in order to incorporate older published shorter sequence datasets). This should be stated here in the Introduction, and form the basis for the new nomenclature for the shorter haplotypes. **R:** This information has been incorporated into the Introduction, and the nomenclature for shorter haplotypes was revised accordingly to follow the approach proposed by Toha et al. (2025).

**Methods**

- **Line 133-134:** I would highly recommend using the numerical system for the shorter haplotype nomenclature. There are several reasons for doing this, primarily to integrate longer and shorter haplotype names in a more intuitive way that reflects equivalent variants (see comment above re. Toha et al. 2025). Also, it does not limit the number of haplotypes to 26. To date there are a total of 28 unique haplotypes, including the ones identified in this manuscript, and likely to be more into the future.

**R:** We thank the reviewer for this recommendation and agree. The nomenclature for shorter haplotypes has been revised to adopt the numerical system, following Toha et al. (2025). This change allows a more intuitive integration between short and long sequence haplotypes and avoids limitations associated with alphabetic naming.

- **Line 135**: Provide more detail on how new names/numbers were chosen. The current designations established by Dutton et al. (2013) and in subsequent designations follow a sequential numbering, but there are some “gaps” that are not yet publicly available on GenBank (e.g. Dc15.1, Dc16.1, Dc21.1, etc…see below). While I appreciate that the authors would not have been aware of unpublished haplotypes assigned as part of informal working group haplotype “clearing houses”, it is important to harmonize the new name designations in this paper, with those currently being used by others with manuscripts in review and being submitted on GenBank, to avoid adding further confusion. I am happy to provide additional GenBank sequences for the authors to incorporate into this paper.

**R:** We thank the reviewer for this important and constructive comment. In the revised manuscript, we have clarified the criteria used for assigning new haplotype names, following a sequential numerical system consistent with Dutton et al. (2013) and subsequent studies, while avoiding conflicts with previously designated haplotypes. We note that some of the previously existing gaps in the numbering system corresponded to haplotypes that were unpublished at the time of our initial analysis but have since been formally published. Accordingly, we have updated the nomenclature to incorporate these newly available data and ensure consistency with current designations. As a result, the haplotype naming system has been revised to improve harmonization with existing and recently published datasets, reducing the risk of duplication or ambiguity. We agree that coordination among research groups is essential to maintain consistency, and we appreciate the reviewer’s willingness to share additional sequences.

- **Line 190-191**: Elaborate on what was the demarcation between the Pacific and Indian Oceans, given the biogeographic complexity related to leatherback nesting sites in the Asia-Pacific and Indo-Pacific region, and the hypothesis (by Dutton et al. 1999 and others) that the Indo-Western-Pacific may have been the source of post-Pleistocene radiation/recolonization to the eastern Pacific. Given the spotty data for the Indo-Western-Pacific, perhaps it doesn’t really matter if the West Pacific and East Pacific are lumped together, but it would be good to clarify and consider how, if at all, parsing out these broad regions, might influence results of the phylogenic analyses and inference of ancestral states. Perhaps food for thought in the Discussion?

**R:** We thank the reviewer for this thoughtful and insightful comment. In the revised manuscript, we have clarified the criteria used to define oceanic regions in the ancestral area reconstruction. Specifically, haplotypes were assigned to Pacific, Atlantic, and Indian Ocean regions based on the geographic designations provided in the original studies, without imposing a strict biogeographic boundary between the Pacific and Indian Oceans, particularly in the Indo-Western-Pacific region where such limits are inherently complex.

- Also note that the geographic location designations for some Pacific and Atlantic datasets are incorrect (see comment about Table S1 for details). This calls into question the validity of the phylogeographic analyses.

**R:** Done. Thanks!

**Results**

- **Line 202:** All but one of the transversions disappear once the erroneous Dc4.2 and 4.3 haplotypes are removed. The only transversion is T-G introduced by the newly assigned haplotypes at position 216 in this study (Table 1). Table 1 also indicates a transversion for Dc20.1 at this position (which is position 230 in the Dutton et al. 2013 Dc1.1 alignment). However, the original published Dc20.1 Toha et al. 2025 did not report this variable site, so something seems to be amiss. I have not had time to double check the sequences for the original CmP20.1, but the T-G transversion here should be confirmed by the authors to rule out alignment errors or typos in the table.

**R:** We thank the reviewer for carefully examining this aspect of the dataset. Following this comment, we thoroughly rechecked all sequence alignments and the corresponding haplotype table. The alignment and Table 1 were carefully revised and are now consistent. The transversion at position 216 (corresponding to position 230 in Dutton et al. 2013) was confirmed based on the sequences included in our dataset, and no alignment errors or typographical inconsistencies were identified. We note, however, that there was some confusion regarding the occurrence of this transversion due to differences in haplotype nomenclature across studies. Specifically, this T–G transversion is present in haplotypes Dc15.1 (corresponding to C3 in Molfetti et al. 2013), Dc16.1 (A5 in Molfetti et al. 2013), and in Dc21.1 (H2), Dc22.1 (H3), and Dc23.1 (H4) as reported by Castillo et al. (2023). Importantly, the haplotype previously labeled as Dc20.1 in an earlier version of our manuscript corresponds to C3 from Molfetti et al. (2013), which we now standardize and propose as Dc15.1 in the revised version. The haplotype Dc20.1, as published by Toha et al. (2025), was incorporated during the final stages of our manuscript preparation. Because haplotype nomenclature in leatherback turtles is continuously being updated and standardized across studies, and given that Toha et al. (2025) was published while our manuscript was under review, this led to a cascading effect in haplotype name assignments. We have now carefully cross-checked and harmonized haplotype identities and names across all referenced datasets to avoid further inconsistencies.

- **Lines 233-236:** These new assigned haplotype names duplicate others that have been informally assigned as part of unpublished work-in-progress (see comments above in methods). **R:** We thank the reviewer for this important observation. We acknowledge that, at the time of our initial analyses, some haplotypes assigned in ongoing or unpublished studies were not yet publicly available, which may have led to potential duplication in nomenclature. Since then, these studies have been published, and we have updated our dataset and haplotype nomenclature accordingly. The revised manuscript reflects these updates, ensuring consistency with currently available data and minimizing the risk of duplication or ambiguity.
- See below for information on each of the specific haplotypes, and suggestions for harmonizing the haplotype names:
  - Note that Dc1.5 was informally assigned, but subsequently determined to be an error, therefore we skipped that number. **R:** We thank the reviewer for this clarification. Dc1.5 was removed from the analysis as it was determined to be erroneous, and the gap in numbering is now explicitly acknowledged in the revised manuscript to avoid confusion.
  - Dc4.2 and Dc4.3 are not valid haplotypes (Maslim et al. 2016) and should be removed, and highlight here for the record that they should not be used. These names were assigned arbitrarily by the author before he was aware of basic best practices for designating new haplotype names. Cite Toha et al (2025) where this point is made (per pers. Comm from the original author, Maslim). Maslim has since redone his study, and completed a comprehensive analysis of these and other Sumatra nesting sites, and confirmed that these haplotypes do not exist (As-singkily et al. in review). Removal of these two sequences will eliminate many of the new variable sites identified in Tables 1 & 2, and errors introduced in the phylogenic analysis. **R:** We thank the reviewer for this important clarification. Dc4.2 and Dc4.3 were removed from the dataset, as these haplotypes are not valid. All analyses, including alignments, haplotype tables, and phylogenetic reconstructions, were redone accordingly. We now explicitly state in the manuscript that these haplotypes should not be used, following recent evaluations (e.g., Toha et al., 2025). The removal of these sequences also resolved spurious variable sites previously identified in Tables 1 and 2 and eliminated associated artifacts in the phylogenetic analyses.
  - Dc16.1 – this appears to be a newly assigned haplotype from a sequence extracted from the whole mitogenome published by Duchene et al (2012). It is unclear why it was designated this number. Dc16.1 has been informally assigned to a sequence from a stranded leatherback in the NE Atlantic (e.g. an orphan haplotype) (LaCasella et al. unpublished). I am happy to provide the sequence and release the GenBank ID if the authors are willing to revise their Dc numbering assignments to the new haplotypes they have identified. Note that the sequence the authors propose to assign to Dc16.1 (681bp), would be more appropriately assigned as a variant of Dc4. Since Dc4.2 and 4.3 are not valid haplotypes, but in the literature, I would suggest assigning it Dc4.4 to avoid future confusion. See comment below regarding shorter 473bp haplotype names that should be harmonized to the base Dc1 -aligned names. In this case the proposed Dc4.4 (681bp) would be the equivalent Dc4 (473bp), as established in Toha et al. (2025). **R:** We thank the reviewer for this detailed and constructive suggestion. Following this recommendation, we revised the designation of this haplotype and reassigned it as Dc4.4, reflecting its relationship with the Dc4 lineage and avoiding potential conflicts with previously assigned or informally designated haplotypes. This change also ensures consistency with the framework proposed by Toha et al. (2025) and improves the correspondence between long and short sequence haplotypes in the revised manuscript.
- Similarily, Dc15.1 was informally assigned, but not published, since it represents another rare orphan haplotype found in a handful of NE Atlantic foraging and stranded turtles. Again, I’m happy to provide the sequence and Genbank ID for the authors to use in this paper. Dc21.1-24.1 are new assignments (of haplotypes originally published under different nomenclature) in this manuscript. These have all been informally assigned to current unpublished haplotypes. If the authors are willing to coordinate, I would be happy to provide the sequences to haplotypes we have assigned as Dc21.1, Dc22.1, Dc23.1 and Dc24.1, to include them in the list. Ideally, the authors would be willing to rename their new haplotypes Dc25.1-Dc28.1, and insert the ones we have already designated, since two of the haplotypes are part of manuscripts currently in review. Our working list only goes through Dc24.1, so this paper would be a timely extension of all the current work in progress. **R:** We thank the reviewer for these valuable clarifications and for the generous offer to share additional unpublished sequences. During the revision process, our primary focus was on identifying and correcting erroneous haplotypes (e.g., Dc4.2 and Dc4.3), rechecking all sequence alignments, and reanalyzing the dataset accordingly. This extensive effort required a complete reassessment of the nomenclature and associated analyses. As a result, although we recognize the importance of harmonizing nomenclature with ongoing work, sequences that are not yet formally published or available in GenBank were not incorporated into the present study. Consequently, we maintained the revised nomenclature based on publicly available data to ensure consistency, transparency, and reproducibility of the analyses. We agree that coordination across research groups is essential, and we see this as an excellent opportunity for future work. The forthcoming publication of these haplotypes will be a valuable contribution and can be readily integrated into the standardized framework proposed here, further strengthening its utility as a reference for the field.
- **Table caption (line 250-261)**: The names in bold represent both previously assigned and the newly assigned haplotypes. This is confusing, please distinguish the current published and assigned **Dc*x.n*** haplotyes from the ones being assigned new names. **R:** We thank the reviewer for this important observation. We agree that the previous use of bold formatting was ambiguous. In the revised manuscript, we clarified the table structure and legend to clearly distinguish between previously published haplotypes and newly assigned names. Standardized haplotype names are now shown in bold, and newly assigned haplotypes are explicitly indicated, improving clarity and avoiding confusion.
- **Lines 279-281:** This is a good point, since we would not expect this stranded turtle to have originated from the Pacific, thus demonstrating the utility of the phylogenetic analysis in this case. **R:** We thank the reviewer for this positive comment. We have clarified this point in the revised manuscript to better highlight the utility of the phylogenetic analysis in inferring the likely origin of stranded individuals.
- **Supplementary Table S1:** Some of the locations are incorrect. ACR in Dutton et al. 2013 refers to Atlantic Costa Rica (Gandoca and Tortuguero), but it is listed under the Pacific locations and combined with the sample from Ostional (Pacific) from Duchene et al. 2012 in both the longer and shorter sequence tables. The shorter sequence table has ACR (Tortuguero and Gandoca) correctly under the Atlantic for the data from Dutton et al. 1999, but incorrectly for the Dutton et al. 2013. This brings into question the accuracy of the geographic assignments for the phylogentic analyses, especially if Caribbean data was conflated with eastern Pacific. **R:** We thank the reviewer for identifying this important error. We corrected the geographic assignments for all ACR samples in Supplementary Table S1, ensuring that Atlantic Costa Rica (Gandoca and Tortuguero) and Pacific Costa Rica (Ostional) are accurately separated in both the long- and short-sequence datasets. We also rechecked the full dataset for consistency of geographic assignments and updated the phylogenetic and phylogeographic analyses accordingly.

**Discussion**

- The Discussion should be revised depending on the outcome of removal of Dc4.2 and 4.3 and correction of the geographic location errors noted above, particularly the paragraphs from lines 438-452. **R:** We thank the reviewer for this important suggestion. The Discussion was revised accordingly, particularly the indicated paragraphs, to reflect the removal of Dc4.2 and Dc4.3 and the corrected geographic assignments. All interpretations were updated to ensure consistency with the revised dataset and analyses.
- **Line 358:** “…this also highlights significant knowledge gaps regarding genetic diversity and population structure within these oceanic regions…” This is true, work in progress is uncovering new diversity (e.g. As-singkily et al. in review), but not at the deep level stated in lines 359-362. **R:** We thank the reviewer for this important clarification. We agree that, although important knowledge gaps remain, the previous text overstated the depth of divergence in this region. In the revised manuscript, we tempered this interpretation and removed statements implying unusually deep genetic differentiation, so that the Discussion now more accurately reflects the currently supported level of diversity and population structure.
- **Line 370:** Pointing out potential NUMTs is good, there is a need to expand awareness. I’m not sure it explains the errors in the case of the Dc4.2 and Dc4.3 study though. Can the sequence be checked against the known NUMT? Unless you can show these are NUMTs, I’d suggest re-organizing this section to include a paragraph addressing caveats of using poor quality data and also point out the sequences that you ended up discarding (mentioned in methods) because of data quality issues. That would allow you highlight some best practices that you use, and include screening for NUMTs as one additional QA/QC step. Otherwise, there are some excellent general points presented in thoughtful ways. Some points however could benefit from more specificity. **R:** We thank the reviewer for this thoughtful and constructive suggestion. We agree that, in the case of Dc4.2 and Dc4.3, there is no clear evidence that these sequences represent NUMTs. Accordingly, we revised this section to avoid attributing these errors to NUMTs and instead emphasize issues related to sequence quality and validation. In the revised manuscript, we reorganized this part of the Discussion to explicitly address data quality caveats, including the identification and removal of problematic sequences (e.g., Dc4.2 and Dc4.3) as part of our quality control procedures. We also highlight best practices for haplotype designation, including careful sequence validation, alignment checking, and screening for potential NUMTs as an additional QA/QC step.
- **Line 381**: points out the value of establishing a more cohesive system with standardized nomenclature. This would be a good place to mention examples with other species (e.g. the Jensen et al 2018 green turtle and Jensen et al. 2025, Shamblin et al 2017)…that illustrate nomenclatures that reflect intuitive internal cohesion between shorter and longer haplotypes by numbering variants of base shorter haplotypes with a longer sequence variant extension (e.g for CmP4 shorter-CmP4.1, 4.2, 4.3, or for CmA5 shorter-CmA5.1, CmA5.2, etc), and reinforce the preference to use a similar approach for leatherbacks. Again, the use of alphabetical haplotypes for shorter sequences does not offer the same level of cohesion. **R:** We thank the reviewer for this valuable suggestion. In the revised manuscript, we incorporated in the Introduction section this point to include examples from other sea turtle species (e.g., Jensen et al., 2019; Shamblin et al., 2017), highlighting nomenclature systems that integrate shorter and longer haplotypes through a hierarchical numerical framework. We also revised our approach accordingly, adopting a numerical system for shorter haplotypes to improve internal consistency and alignment with these established practices.
- **Line 350:** “our study clarifies the relationship between foraging areas and nesting populations”- it is not clear how the analysis in this manuscript does this…be more specific if possible. Also, a related point in Lines 385-389: this seems like a general vague statement that repeats common statements from other more targeted MSA studies that have already established the connectivity between Brazil (and other foraging grounds in Uruguay and Argentina and nesting populations in West Africa). What additional contribution does this study make? The presence of shared common haplotypes by itself doesn’t necessarily indicate connectivity, but perhaps mentioning that to supplement these MSA studies, the more comprehensive phylogenetic analysis that includes orphan haplotypes can at least help rule out some distant ocean basin origin, as your findings for Dc1.7 illustrate. **R:** We thank the reviewer for this important and constructive comment. We agree that the previous text overstated our contribution and did not clearly explain how our analysis supports this statement. In the revised manuscript, we have rephrased this section to avoid implying direct inference of connectivity and to better define the scope of our results. Specifically, we now clarify that our contribution is complementary to mixed-stock analyses (MSA). Rather than directly inferring connectivity, our phylogenetic framework provides additional context by incorporating orphan haplotypes and placing them within a global evolutionary framework. For example, the phylogenetic placement of Dc1.7 within an Atlantic-associated lineage helps constrain its likely origin and rule out distant ocean basin sources. We also revised the related section (Lines 385–389) to remove generalized statements and avoid repetition of conclusions already established by MSA studies. The revised text now more clearly highlights the specific contribution of our study in refining hypotheses about the origin of individuals with unknown nesting provenance.
- **Similarly line 388-389**: “This highlights the need for coordinated protection measures, particularly in reducing bycatch, and pollution in key migratory corridors” is an obvious often-repeated general statement, but are there specific actions that can be given? While MSA and regional connectivity studies address relatively shorter-term management timescales, identifying synonymous haplotypes on a broad global scale seems be the more relevant application from this study, with management implications on deeper evolutionary time scales (climate change, etc). You could discuss what, if any, the implications are of your global analysis results. **R:** We thank the reviewer for this important comment and agree that the previous statement was overly general. In the revised manuscript, we replaced this section with a more specific discussion that distinguishes short-term management applications from the broader, long-term implications of our global phylogenetic framework. We now explicitly emphasize that the primary contribution of this study lies in providing a standardized global framework for interpreting haplotype diversity across ocean basins. This approach is particularly relevant for long-term conservation planning, as it improves the integration of datasets across regions, refines historical connectivity inferences, and establishes a robust baseline for detecting future changes in distribution patterns, especially under climate-driven scenarios.

**Please do not hesitate to contact me with any further comments or if you require additional clarification. I sincerely appreciate your and the reviewers’ valuable feedback and look forward to your response.**

**Sincerely,**

**Dr. Wesley D. Colombo**

**Dr. Sarah M. Vargas**
