# Supplementary material for: Haplotypes across the Oceans: worldwide phylogeography, evolution, conservation and nomenclature standardization in the leatherback sea turtle (Dermochelys coriacea)
Source: PLoS One. 2026 Aug 19;21(8):e0354151. doi: 10.1371/journal.pone.0354151 (PMC13489440; doi:10.1371/journal.pone.0354151)
Supplement: Supplementary file 3 [file pone.0354151.s008.docx]

**Dear Dr. Ulrich Joger,**

**We hope this message finds you well. Thank you for your positive assessment of our manuscript, “*Haplotypes across the Oceans: worldwide phylogeography, evolution, conservation and nomenclature standardization in the leatherback sea turtle (Dermochelys coriacea)*”, and for the opportunity to revise it further. We are pleased to submit the revised version of the manuscript, in which we carefully considered and addressed all comments and suggestions from the reviewers and editor, as detailed below. Following the journal’s instructions, we have uploaded both a clean version of the revised manuscript and a marked version highlighting all changes. We sincerely appreciate the time and effort invested in evaluating our work and believe that the revisions have substantially improved the manuscript.**

**Editor Comments:**

**Thank you for obeying the reviewer's wishes. Now he only has a few additional points. If you incorporate them, the paper can be published.**

**R:** We thank the Editor for the positive evaluation of our revised manuscript and for the opportunity to submit a further revision. We carefully addressed all comments and suggestions raised during the second round of review. In particular, we incorporated the additional points requested by the reviewer, including the evaluation and citation of the recently published paper by Jensen et al. (2026), which we considered highly relevant to the objectives of our study. The Discussion section was revised accordingly, and the reference list was updated to include this publication. In addition, we conducted a thorough review of the entire manuscript and reference list. This process allowed us to identify and correct several minor typographical, formatting, nomenclatural, and reference-related inconsistencies. All modifications are documented in the tracked-changes version submitted alongside the clean, revised manuscript. We believe that these revisions have further improved the clarity, accuracy, and overall quality of the manuscript, and we sincerely appreciate the Editor’s and the reviewer’s valuable guidance throughout the review process.

**Reviewer 1:** *Peter Dutton*

**General comments:**

Thank you for your comprehensive revision of the manuscript and your thorough and thoughtful responses to all my comments and suggestions. This is now a tremendously relevant, useful and timely paper that will serve as a great basis for ongoing and future leatherback mtDNA studies. I appreciate the tremendous amount of work that you have put in to correct data errors and redo the phylogenetic analyses. I have only minor comments and suggestions, below:

I am sorry the authors did not want to incorporate the unpublished haplotype names into this manuscript, but understand their reluctance to wait for the relevant studies to be published, and to confine their paper to sequences that were publicly available on GenBank at the time of their analysis. To be clear, I was not necessarily suggesting the sequences be incorporated into their analysis at this point, but simply re-numbering their new Dc assignment names, and noting the current ID numbers that have been independently assigned in their list to avoid the duplication. This is more of a missed opportunity, so it remains a soft suggestion from me, rather than requirement. If published as is, the current working group databases and previous GenBank submissions (that are now publicly available) will need to be adjusted to remove the duplicate haplotype numbers, but that’s OK, our relevant Genbank records for Dc15.1, 16.1,21.1 and 22.1 can be updated fairly quickly, so that they are harmonized with the names assigned in Table 3. Timing is unfortunate with our two parallel endeavors to standardize nomenclature being published at the same time, however they are complementary, and the Jensen et al. points to the dynamic database, not a fixed list. I respectfully ask that the authors cite the database paper, that has now been provisionally accepted, and suggest:

Please add a sentence to the Discussion to recognize the online mtDNA website that allows dynamic updating and standardized mtDNA nomenclature, and cite the publication below (Jensen et al. 2026). This is exactly the resource that the current manuscript points out is missing and citing it will point the reader to current/updated resources for coordinating mtDNA haplotype nomenclature into the future, and operationalize the updated work that the authors have done in their study for leatherbacks; we will incorporate the information into the leatherback working data base, which will be publicly available as noted in the Jensen et al. paper.

Jensen, M.P., Frankham, G.J., O’Friel, C.A., LaCasella, E.L., Morgan, K.I., Sola. M., Webster, L.M.I., Dutton, P.H. & Madden, C.A. (2026). ShellBank: traceability toolkit and global database of marine turtle DNA. Frontiers in Marine Science (in press).

I previously discussed this database with the authors, and have now separately shared a copy of the final revised version that is currently in process.

Line 402-3 might be the most appropriate place to refer to this database: “Standardizing haplotype classification enhances the clarity of genetic connectivity patterns, enabling more reliable conservation assessments and facilitating collaboration across research groups…” Perhaps add a sentence: “…Recently an open-access sea turtle mtDNA database was established to provide a resource for standardizing haplotype nomenclature (Jensen et al. 2026). Information from our study will provide a verified dataset for leatherbacks that includes all the published to date, which can then be updated dynamically in the future, thus enhancing standardization and collaboration between different research groups…” or something like this.

**R:** We sincerely thank Dr. Peter H. Dutton for his very positive assessment of our revised manuscript and for his thoughtful and constructive comments throughout the review process. We greatly appreciate his recognition of the effort invested in correcting data inconsistencies, revisiting the haplotype nomenclature, and re-evaluating the phylogenetic analyses. His expertise and suggestions have substantially improved the quality, clarity, and relevance of this study. We also appreciate the reviewer’s perspective regarding the unpublished haplotype names and understand the value of avoiding future duplication of nomenclatural assignments. As noted previously, our decision to restrict the analysis and nomenclature framework to sequences that were publicly available in GenBank at the time of the study was made to ensure transparency, reproducibility, and long-term traceability of the proposed nomenclature. More specifically, our dataset was designed to include all *D. coriacea* mtDNA control region sequences publicly available in GenBank up to 1 February 2026, providing a clearly defined and reproducible temporal framework for the analyses. Nevertheless, we agree that the parallel efforts to standardize leatherback turtle haplotypes are complementary and will ultimately contribute to a more robust and unified framework for future studies. Following the reviewer’s recommendation, we carefully evaluated the recently published paper by Jensen et al. (2026) and concluded that it represents a highly relevant contribution to the field. The ShellBank initiative directly addresses one of the key challenges highlighted in our manuscript by providing an open-access and dynamically updated resource for marine turtle mtDNA nomenclature and data sharing. Accordingly, we incorporated the suggested citation into the Discussion section, adding a paragraph recognizing the importance of this database for future harmonization of leatherback turtle haplotype nomenclature and the continued integration of genetic information across studies. We also emphasize that the standardized dataset presented in our study provides a comprehensive and validated baseline that can be incorporated into and updated through this international framework. In addition, a careful review of the entire manuscript was conducted following this second round of revisions. Minor typographical, formatting, nomenclatural, and reference-related inconsistencies were corrected, and the reference list was updated to include Jensen et al. (2026). All modifications are documented in the tracked-changes version of the manuscript. We are grateful to the reviewer for drawing our attention to this important new resource and for his continued support and guidance throughout the review process.

Finally, a couple minor wording suggestions:

1) line 204 of revised mss- ”.. The delimitation between the Pacific and Indian Oceans..”, say “demarcation” instead of “delimitation” for better english usage. **R:** Done.
2) Line 473: “In conclusion, the haplotype nomenclature standardization proposed in this study”- change “proposed” to “adopted” (This study adopts and builds on nomenclature frameworks proposed in previous studies as described throughout the manuscript). **R:** Done.

**Please do not hesitate to contact me with any further comments or if you require additional clarification. I sincerely appreciate your and the reviewers’ valuable feedback and look forward to your response.**

**Sincerely,**

**Dr. Wesley D. Colombo**

**Dr. Sarah M. Vargas**
