# Supplementary material for: Haplotypes across the Oceans: worldwide phylogeography, evolution, conservation and nomenclature standardization in the leatherback sea turtle (Dermochelys coriacea)
Source: PLoS One. 2026 Aug 19;21(8):e0354151. doi: 10.1371/journal.pone.0354151 (PMC13489440; doi:10.1371/journal.pone.0354151)
Supplement: Peer review history

# Peer review: Decision Letter 0

**DOI:** 10.1371/journal.pone.0354151.r001

**Authors:** Ulrich Joger

3 Nov 2025

PONE-D-25-32257

Haplotypes across the Oceans: worldwide phylogeography, evolution, conservation and nomenclature standardization in the leatherback sea turtle (Dermochelys coriacea)

PLOS ONE

Dear Dr. Vargas,

Thank you for submitting your manuscript to PLOS ONE. After careful consideration, we feel that it has merit but does not fully meet PLOS ONE’s publication criteria as it currently stands. Therefore, we invite you to submit a revised version of the manuscript that addresses the points raised during the review process.

- **Your manuscript was judged "major revision necessary".**

Please submit your revised manuscript by Dec 17 2025 11:59PM. If you will need more time than this to complete your revisions, please reply to this message or contact the journal office at plosone@plos.org. When you're ready to submit your revision, log on to https://www.editorialmanager.com/pone/ and select the 'Submissions Needing Revision' folder to locate your manuscript file.

Please include the following items when submitting your revised manuscript:

- A rebuttal letter that responds to each point raised by the academic editor and reviewer(s). You should upload this letter as a separate file labeled 'Response to Reviewers'.
- A marked-up copy of your manuscript that highlights changes made to the original version. You should upload this as a separate file labeled 'Revised Manuscript with Track Changes'.
- An unmarked version of your revised paper without tracked changes. You should upload this as a separate file labeled 'Manuscript'.

If you would like to make changes to your financial disclosure, please include your updated statement in your cover letter. Guidelines for resubmitting your figure files are available below the reviewer comments at the end of this letter.

If applicable, we recommend that you deposit your laboratory protocols in protocols.io to enhance the reproducibility of your results. Protocols.io assigns your protocol its own identifier (DOI) so that it can be cited independently in the future. For instructions see: https://journals.plos.org/plosone/s/submission-guidelines#loc-laboratory-protocols. Additionally, PLOS ONE offers an option for publishing peer-reviewed Lab Protocol articles, which describe protocols hosted on protocols.io. Read more information on sharing protocols at https://plos.org/protocols?utm_medium=editorial-email&utm_source=authorletters&utm_campaign=protocols.

We look forward to receiving your revised manuscript.

Kind regards,

Ulrich Joger

Academic Editor

PLOS ONE

**Journal Requirements:**

1. When submitting your revision, we need you to address these additional requirements.

Please ensure that your manuscript meets PLOS ONE's style requirements, including those for file naming. The PLOS ONE style templates can be found at

https://journals.plos.org/plosone/s/file?id=wjVg/PLOSOne_formatting_sample_main_body.pdf and

https://journals.plos.org/plosone/s/file?id=ba62/PLOSOne_formatting_sample_title_authors_affiliations.pdf

2. Thank you for stating the following financial disclosure:

The funding was provided by the Renova Foundation – Brazil (Grant No. 30/2018) through its Technical-Scientific Cooperation Agreement with FEST, which awarded postdoctoral fellowships to WDC and SSAT, a technical support to JLGF, an undergraduate scholarship to GLS, and a research fellowship to SMV.

Please state what role the funders took in the study.  If the funders had no role, please state: "The funders had no role in study design, data collection and analysis, decision to publish, or preparation of the manuscript."

If this statement is not correct you must amend it as needed.

Please include this amended Role of Funder statement in your cover letter; we will change the online submission form on your behalf.

3. PLOS requires an ORCID iD for the corresponding author in Editorial Manager on papers submitted after December 6th, 2016. Please ensure that you have an ORCID iD and that it is validated in Editorial Manager. To do this, go to ‘Update my Information’ (in the upper left-hand corner of the main menu), and click on the Fetch/Validate link next to the ORCID field. This will take you to the ORCID site and allow you to create a new iD or authenticate a pre-existing iD in Editorial Manager.

4. We note that Figure 2 in your submission contain map/satellite images which may be copyrighted. All PLOS content is published under the Creative Commons Attribution License (CC BY 4.0), which means that the manuscript, images, and Supporting Information files will be freely available online, and any third party is permitted to access, download, copy, distribute, and use these materials in any way, even commercially, with proper attribution. For these reasons, we cannot publish previously copyrighted maps or satellite images created using proprietary data, such as Google software (Google Maps, Street View, and Earth). For more information, see our copyright guidelines: http://journals.plos.org/plosone/s/licenses-and-copyright.

We require you to either present written permission from the copyright holder to publish these figures specifically under the CC BY 4.0 license, or remove the figures from your submission:

a. You may seek permission from the original copyright holder of Figure 2 to publish the content specifically under the CC BY 4.0 license.

We recommend that you contact the original copyright holder with the Content Permission Form (http://journals.plos.org/plosone/s/file?id=7c09/content-permission-form.pdf) and the following text:

“I request permission for the open-access journal PLOS ONE to publish XXX under the Creative Commons Attribution License (CCAL) CC BY 4.0 (http://creativecommons.org/licenses/by/4.0/). Please be aware that this license allows unrestricted use and distribution, even commercially, by third parties. Please reply and provide explicit written permission to publish XXX under a CC BY license and complete the attached form.”

Please upload the completed Content Permission Form or other proof of granted permissions as an "Other" file with your submission.

In the figure caption of the copyrighted figure, please include the following text: “Reprinted from [ref] under a CC BY license, with permission from [name of publisher], original copyright [original copyright year].”

b. If you are unable to obtain permission from the original copyright holder to publish these figures under the CC BY 4.0 license or if the copyright holder’s requirements are incompatible with the CC BY 4.0 license, please either i) remove the figure or ii) supply a replacement figure that complies with the CC BY 4.0 license. Please check copyright information on all replacement figures and update the figure caption with source information. If applicable, please specify in the figure caption text when a figure is similar but not identical to the original image and is therefore for illustrative purposes only.

The following resources for replacing copyrighted map figures may be helpful:

USGS National Map Viewer (public domain): http://viewer.nationalmap.gov/viewer/

The Gateway to Astronaut Photography of Earth (public domain): http://eol.jsc.nasa.gov/sseop/clickmap/

Maps at the CIA (public domain): https://www.cia.gov/library/publications/the-world-factbook/index.html and https://www.cia.gov/library/publications/cia-maps-publications/index.html

NASA Earth Observatory (public domain): http://earthobservatory.nasa.gov/

Landsat: http://landsat.visibleearth.nasa.gov/

USGS EROS (Earth Resources Observatory and Science (EROS) Center) (public domain): http://eros.usgs.gov/#

Natural Earth (public domain): http://www.naturalearthdata.com/

5. We are unable to open your Supporting Information file “9-S2_Haplotype-data-file_681bp - OK.fas and 10-S3_Haplotype-data-file_473bp - OK.fas“. Please kindly revise as necessary and re-upload.

6. If the reviewer comments include a recommendation to cite specific previously published works, please review and evaluate these publications to determine whether they are relevant and should be cited. There is no requirement to cite these works unless the editor has indicated otherwise.

**Additional Editor Comments:**

The reviewer made very useful suggestions which you should follow. In particular, the fact that haplotypes 4.2 and 4.3 are erroneous, invalidates part of your study. All calculations involving these haplotypes must be redone.

In addition, statements like:

unique haplotypes in regions like Sumatra, suggests considerable dispersal and migratory potential.

However, this also highlights significant knowledge gaps regarding genetic diversity and population

structure within these oceanic regions [17]. Notably, Sumatra haplotypes (Dc4.2 ≈ DcJ, Dc4.3 ≈ DcM)

show an exceptionally high level of polymorphism and unusually long branch lengths compared to all

other haplotypes analyzed in this study (discussion) must be changed.

[Note: HTML markup is below. Please do not edit.]

Reviewers' comments:

Reviewer's Responses to Questions

**Comments to the Author**

1. Is the manuscript technically sound, and do the data support the conclusions?

The manuscript must describe a technically sound piece of scientific research with data that supports the conclusions. Experiments must have been conducted rigorously, with appropriate controls, replication, and sample sizes. The conclusions must be drawn appropriately based on the data presented.

Reviewer #1: Partly

**********

2. Has the statistical analysis been performed appropriately and rigorously?

Reviewer #1: Yes

**********

3. Have the authors made all data underlying the findings in their manuscript fully available?

The PLOS Data policy requires authors to make all data underlying the findings described in their manuscript fully available without restriction, with rare exception (please refer to the Data Availability Statement in the manuscript PDF file). The data should be provided as part of the manuscript or its supporting information, or deposited to a public repository. For example, in addition to summary statistics, the data points behind means, medians and variance measures should be available. If there are restrictions on publicly sharing data—e.g. participant privacy or use of data from a third party—those must be specified.

Reviewer #1: Yes

**********

4. Is the manuscript presented in an intelligible fashion and written in standard English?

PLOS ONE does not copyedit accepted manuscripts, so the language in submitted articles must be clear, correct, and unambiguous. Any typographical or grammatical errors should be corrected at revision, so please note any specific errors here.

Reviewer #1: Yes

**********

5. Review Comments to the Author

Please use the space provided to explain your answers to the questions above. You may also include additional comments for the author, including concerns about dual publication, research ethics, or publication ethics. (Please upload your review as an attachment if it exceeds 20,000 characters)

**Reviewer #1:**  I checked "partly" to question #1, only because there are errors in the input data (detailed in the attached review). The actual analyses and design was very rigorous, and reflects a high level of technical competence

**********

6. PLOS authors have the option to publish the peer review history of their article (what does this mean?). If published, this will include your full peer review and any attached files.

If you choose “no”, your identity will remain anonymous but your review may still be made public.

**Do you want your identity to be public for this peer review?**  For information about this choice, including consent withdrawal, please see our Privacy Policy.

Reviewer #1: **Yes:** Peter H. Dutton

**********

[NOTE: If reviewer comments were submitted as an attachment file, they will be attached to this email and accessible via the submission site. Please log into your account, locate the manuscript record, and check for the action link "View Attachments". If this link does not appear, there are no attachment files.]

To ensure your figures meet our technical requirements, please review our figure guidelines: https://journals.plos.org/plosone/s/figures

You may also use PLOS’s free figure tool, NAAS, to help you prepare publication quality figures: https://journals.plos.org/plosone/s/figures#loc-tools-for-figure-preparation.

NAAS will assess whether your figures meet our technical requirements by comparing each figure against our figure specifications.

Attachment Submitted filename: Review of PONE-D-25-32257.docx

# Author comment: Author response to Decision Letter 1

**DOI:** 10.1371/journal.pone.0354151.r002

1 Apr 2026

Dear Dr. Ulrich Joger,

We hope this message finds you well. Thank you for your insightful feedback on our manuscript, “Haplotypes across the Oceans: worldwide phylogeography, evolution, conservation and nomenclature standardization in the leatherback sea turtle (Dermochelys coriacea)”. We are pleased to submit the revised version of the manuscript, in which we carefully considered and addressed all the reviewers’ suggestions, including yours, as detailed below. As requested, we have uploaded a clean version of the revised manuscript without tracked changes, as well as a marked version highlighting all modifications.

Editor Comments:

The reviewer made very useful suggestions which you should follow. In particular, the fact that haplotypes 4.2 and 4.3 are erroneous, invalidates part of your study. All calculations involving these haplotypes must be redone. In addition, statements like: “unique haplotypes in regions like Sumatra, suggests considerable dispersal and migratory potential. However, this also highlights significant knowledge gaps regarding genetic diversity and population structure within these oceanic regions [17]. Notably, Sumatra haplotypes (Dc4.2 ≈ DcJ, Dc4.3 ≈ DcM) show an exceptionally high level of polymorphism and unusually long branch lengths compared to all other haplotypes analyzed in this study (discussion) must be changed.

R: We thank the Editor for this important observation and fully agree. Following this recommendation, we removed haplotypes 4.2 and 4.3 from the dataset because they were identified as sequencing errors by the recently published paper, and we revised the manuscript accordingly. This exclusion is now explicitly stated in the Material and Methods section. All analyses affected by these erroneous haplotypes were redone using the corrected dataset, including haplotype alignments, haplotype counts, variable-site assessments, haplotype networks, synonymization tables, and the subsequent phylogeographic interpretation. The revised version now reports 32 haplotypes in the long-sequence dataset and 24 haplotypes in the short-sequence dataset after these corrections. In addition, we removed the discussion statements that interpreted the former Sumatra haplotypes as highly divergent or unusually polymorphic, since those inferences were based on erroneous sequences and are therefore not valid. The revised manuscript no longer uses haplotypes 4.2 and 4.3 to support claims about exceptional polymorphism, long branch lengths, or dispersal potential associated with Sumatra. The remaining discussion was adjusted to reflect only the validated haplotypes and the corrected analyses.

Reviewer 1: Peter Dutton

General comments:

This manuscript addresses an ongoing problem of confusing nomenclature for leatherback turtle Control Region mtDNA haplotypes in the literature, a primary genetic marker for sea turtle population genetic studies. While standardized nomenclature has been in use as proposed by Dutton et al 2013, it’s effective management (e.g. designating names to new haplotypes as they are discovered in a systematic way) and has relied on informal working group networking, which has not been adequate as more researchers and students conduct mtDNA sequencing studies. The problem is further compounded by use of longer sequences that increasingly characterize more recent studies compared with older historic studies based on shorter sequences. With this manuscript, the authors make a very important contribution, by consolidating and analyzing all the previously published sequence data in order to cross reference equivalent haplotypes into a standardized nomenclature. They further conduct a global phylogenetic analysis with this standardized dataset. This is a tremendously useful resource, and overall, a well written paper, however there are some major problems that need to be addressed before this can be published (in its current state, it risks adding further confusion, which undermines the goal of their efforts).

R: We sincerely thank the reviewer for this thoughtful and highly relevant assessment of our manuscript. We greatly appreciate the recognition of the importance and potential utility of our work in addressing the long-standing issue of inconsistent haplotype nomenclature in leatherback turtle mtDNA studies. We fully acknowledge the reviewer’s concern that, in its previous version, the manuscript could inadvertently contribute to further confusion. In response, we have carefully revised the manuscript to ensure clarity, consistency, and robustness of the proposed nomenclature framework and associated analyses.

Specifically, we have:

● Corrected critical data issues, including the removal of erroneous haplotypes (e.g., Dc4.2 and Dc4.3), and recomputed all affected analyses, ensuring that the standardized dataset is accurate and reliable.

● Revised the haplotype cross-referencing system, improving transparency in how previously published haplotypes are matched and synonymized across studies.

● Clarified the criteria used for haplotype designation, particularly in relation to differences between short and long sequence datasets, to avoid ambiguity in future applications.

● Improved the presentation of results and supporting tables, making it easier for readers to trace equivalences among haplotypes and understand how the standardized nomenclature is applied.

● Revised the discussion throughout the manuscript to remove unsupported interpretations and ensure that all conclusions are strictly based on validated data.

We believe these revisions substantially reduce the risk of misinterpretation and significantly strengthen the manuscript’s contribution as a reference framework for future studies. We are grateful for the reviewer’s insights, which have helped us improve both the rigor and clarity of the work.

Major issues:

The authors have

1) missed an opportunity to critically evaluate at least one study that introduces significant errors, and thus by including Dc4.2 and Dc4.3 (Maslim et al. 2016) undermines the validity of the network and phylogenetic analysis. See below for details. R: We thank the reviewer for this important comment and fully agree that the inclusion of Dc4.2 and Dc4.3, as reported by Maslim et al. (2016), compromised the interpretation of part of our original analyses. In the revised manuscript, we addressed this issue explicitly and critically. First, we removed Dc4.2 and Dc4.3 from the dataset after recognizing that these haplotypes were erroneous and should not be retained in the standardized nomenclature framework. As a consequence, all analyses affected by their inclusion were redone using the corrected dataset, including the haplotype network, phylogenetic reconstruction, and all related summary calculations. Second, we revised the text to make clear that the previous interpretation associated with these haplotypes was invalid. We removed statements suggesting exceptional divergence, long branch lengths, or unusual polymorphism associated with the Sumatra sequences, since these inferences were based on erroneous haplotypes and were therefore unsupported. Third, we agree that this was an important opportunity to more critically assess problematic records in the literature. Accordingly, the revised manuscript now adopts a more cautious and critical approach when consolidating published haplotypes, emphasizing that literature-derived sequence records must be carefully validated before being incorporated into standardized reference frameworks. We appreciate the reviewer for highlighting this issue, which has significantly improved the rigor, accuracy, and reliability of the revised manuscript.

2) incorrectly incorporated an Atlantic dataset into the Pacific dataset, further undermining the validity of the phylogeographic analyses. R: We thank the reviewer for this important clarification and fully agree with this assessment. In the revised manuscript, we have explicitly addressed these issues.

3) failed to incorporate in progress and new haplotypes that were not available on GenBank at the time their analysis was done, thus leading to duplication of “new” haplotype designations. This is understandable and justifiable, but will lead to even more confusion if not remedied. I have provided details and suggestions below, with the intent of encouraging a more up-to-date paper that could be a tremendously useful resource. R: We thank the reviewer for this important and constructive comment. We agree that the dynamic nature of haplotype discovery represents a major challenge for maintaining a consistent and up-to-date nomenclature framework. At the time our analyses were originally conducted, all available sequence data deposited in GenBank and accessible through the literature were included. However, as correctly noted by the reviewer, it is inherently difficult to account for ongoing or unpublished studies at the time of data compilation, which may lead to overlapping or duplicated haplotype designations. Importantly, between the previous version of the manuscript and this resubmission, additional haplotypes have been formally published, and previously included erroneous haplotypes (e.g., Dc4.2 and Dc4.3) have been removed from our dataset. In response, we have updated the dataset and revised the nomenclature framework accordingly. We reorganized the haplotype designation system to incorporate these newly published haplotypes and to avoid duplication or ambiguity in naming. The revised manuscript now reflects an updated and consolidated nomenclature that accounts for both the corrected dataset and the most recent available information. We fully agree with the reviewer that maintaining consistency in haplotype naming requires continuous updates, and we emphasize in the revised manuscript the need for coordinated efforts and centralized curation to minimize future inconsistencies.

4) The proposed alphabetic nomenclature for the shorter sequences is problematic and counter to a more generally adopted approach adopted for other sea turtle species, including recently for leatherbacks (Toha et al. 2025). R: We thank the reviewer for this important point and agree that consistency with existing nomenclature systems is essential. In the revised manuscript, we replaced the alphabetic nomenclature for shorter sequences with the numerical system proposed by Toha et al. (2025), ensuring alignment with current standards and improving compatibility across studies.

5) The authors could broaden the Introduction to frame the topic of inconsistent nomenclature to other species of sea turtles by including other papers that are relevant, particularly:

a) Jensen et al. 2019. The evolutionary history and global phylogeography of the green turtle (Chelonia mydas). J. Biogeogr., 46(5), pp.860-870. doi.org/10.1111/jbi.13483

b) Shamblin et al 2017. Mexican origins for the Texas green turtle foraging aggregation: A cautionary tale of incomplete baselines and poor marker resolution. JEMBE 488: 111-120. https://doi.org/10.1016/j.jembe.2016.11.009 [green turtles, uses haplotype nomenclature that reconciles shorter and longer sequence data].

c) Shamblin et al. 2014. PLoS ONE 9(1): e85956. doi:10.1371/ journal.pone.0085956 (Loggerhead turtles). [This paper incorporates shorter and longer mtDNA sequence data as part of a Working Group to coordinate nomenclature among different research groups].

There are other examples, but these are particularly relevant. Below, I detail out comments, concerns and suggestions, including minor suggested edits.

R: We thank the reviewer for this valuable suggestion. The recommended references (Jensen et al., 2019; Shamblin et al., 2017; Shamblin et al., 2014) have been incorporated into the Introduction to broaden the context and highlight that challenges related to inconsistent haplotype nomenclature and sequence length integration are not unique to leatherback turtles, but are also recognized in other sea turtle species.

Abstract

● Line 27: replace “known” with “published” or “publicly available”. R: Done.

● Line 57: from this point on it is preferable to refer to “leatherback turtles” rather than “leatherback sea turtles”. R: Done. We have replaced “leatherback sea turtles” with “leatherback turtles” throughout the manuscript.

● Lines 71-72: Toha et al. (2025) moved away from the older alphabetical haplotype nomenclature to adopt more informative nomenclature that reconciles the longer sequence haplotypes with corresponding shorter haplotypes (in order to incorporate older published shorter sequence datasets). This should be stated here in the Introduction, and form the basis for the new nomenclature for the shorter haplotypes. R: This information has been incorporated into the Introduction, and the nomenclature for shorter haplotypes was revised accordingly to follow the approach proposed by Toha et al. (2025).

Methods

● Line 133-134: I would highly recommend using the numerical system for the shorter haplotype nomenclature. There are several reasons for doing this, primarily to integrate longer and shorter haplotype names in a more intuitive way that reflects equivalent variants (see comment above re. Toha et al. 2025). Also, it does not limit the number of haplotypes to 26. To date there are a total of 28 unique haplotypes, including the ones identified in this manuscript, and likely to be more into the future.

R: We thank the reviewer for this recommendation and agree. The nomenclature for shorter haplotypes has been revised to adopt the numerical system, following Toha et al. (2025). This change allows a more intuitive integration between short and long sequence haplotypes and avoids limitations associated with alphabetic naming.

● Line 135: Provide more detail on how new names/numbers were chosen. The current designations established by Dutton et al. (2013) and in subsequent designations follow a sequential numbering, but there are some “gaps” that are not yet publicly available on GenBank (e.g. Dc15.1, Dc16.1, Dc21.1, etc…see below). While I appreciate that the authors would not have been aware of unpublished haplotypes assigned as part of informal working group haplotype “clearing houses”, it is important to harmonize the new name designations in this paper, with those currently being used by others with manuscripts in review and being submitted on GenBank, to avoid adding further confusion. I am happy to provide additional GenBank sequences for the authors to incorporate into this paper.

R: We thank the reviewer for this important and constructive comment. In the revised manuscript, we have clarified the criteria used for assigning new haplotype names, following a sequential numerical system consistent with Dutton et al. (2013) and subsequent studies, while avoiding conflicts with previously designated haplotypes. We note that some of the previously existing gaps in the numbering system corresponded to haplotypes that were unpublished at the time of our initial analysis but have since been formally published. Accordingly, we have updated the nomenclature to incorporate these newly available data and ensure consistency with current designations. As a result, the haplotype naming system has been revised to improve harmonization with existing and recently published datasets, reducing the risk of duplication or ambiguity. We agree that coordination among research groups is essential to maintain consistency, and we appreciate the reviewer’s willingness to share additional sequences.

● Line 190-191: Elaborate on what was the demarcation between the Pacific and Indian Oceans, given the biogeographic complexity related to leatherback nesting sites in the Asia-Pacific and Indo-Pacific region, and the hypothesis (by Dutton et al. 1999 and others) that the Indo-Western-Pacific may have been the source of post-Pleistocene radiation/recolonization to the eastern Pacific. Given the spotty data for the Indo-Western-Pacific, perhaps it doesn’t really matter if the West Pacific and East Pacific are lumped together, but it would be good to clarify and consider how, if at all, parsing out these broad regions, might influence results of the phylogenic analyses and inference of ancestral states. Perhaps food for thought in the Discussion?

R: We thank the reviewe

Attachment Submitted filename: 12-Response to Reviewers_Colombo-etal.docx

# Peer review: Decision Letter 1

**DOI:** 10.1371/journal.pone.0354151.r003

**Authors:** Ulrich Joger

5 Jun 2026

PONE-D-25-32257R1

Haplotypes across the Oceans: worldwide phylogeography, evolution, conservation and nomenclature standardization in the leatherback sea turtle (Dermochelys coriacea)

PLOS One

Dear Dr. Vargas,

Thank you for submitting your manuscript to PLOS ONE. After careful consideration, we feel that it has merit but does not fully meet PLOS ONE’s publication criteria as it currently stands. Therefore, we invite you to submit a revised version of the manuscript that addresses the points raised during the review process.

Please submit your revised manuscript by Jul 20 2026 11:59PM. If you will need more time than this to complete your revisions, please reply to this message or contact the journal office at plosone@plos.org. When you're ready to submit your revision, log on to https://www.editorialmanager.com/pone/ and select the 'Submissions Needing Revision' folder to locate your manuscript file.

Please include the following items when submitting your revised manuscript:

- A letter that responds to each point raised by the academic editor and reviewer(s). You should upload this letter as a separate file labeled 'Response to Reviewers'.
- A marked-up copy of your manuscript that highlights changes made to the original version. You should upload this as a separate file labeled 'Revised Manuscript with Track Changes'.
- An unmarked version of your revised paper without tracked changes. You should upload this as a separate file labeled 'Manuscript'.

If you would like to make changes to your financial disclosure, please include your updated statement in your cover letter. Guidelines for resubmitting your figure files are available below the reviewer comments at the end of this letter.

If applicable, we recommend that you deposit your laboratory protocols in protocols.io to enhance the reproducibility of your results. Protocols.io assigns your protocol its own identifier (DOI) so that it can be cited independently in the future. For instructions see: https://journals.plos.org/plosone/s/submission-guidelines#loc-laboratory-protocols. Additionally, PLOS ONE offers an option for publishing peer-reviewed Lab Protocol articles, which describe protocols hosted on protocols.io. Read more information on sharing protocols at https://plos.org/protocols?utm_medium=editorial-email&utm_source=authorletters&utm_campaign=protocols.

As the corresponding author, your ORCID iD is verified in the submission system and will appear in the published article. PLOS supports the use of ORCID, and we encourage all coauthors to register for an ORCID iD and use it as well. Please encourage your coauthors to verify their ORCID iD within the submission system before final acceptance, as unverified ORCID iDs will not appear in the published article. *Only*  the individual author can complete the verification step; PLOS staff *cannot*  verify ORCID iDs on behalf of authors.

We look forward to receiving your revised manuscript.

Kind regards,

Ulrich Joger

Academic Editor

PLOS One

Journal Requirements:

If the reviewer comments include a recommendation to cite specific previously published works, please review and evaluate these publications to determine whether they are relevant and should be cited. There is no requirement to cite these works unless the editor has indicated otherwise.

Please review your reference list to ensure that it is complete and correct. If you have cited papers that have been retracted, please include the rationale for doing so in the manuscript text, or remove these references and replace them with relevant current references. Any changes to the reference list should be mentioned in the rebuttal letter that accompanies your revised manuscript. If you need to cite a retracted article, indicate the article’s retracted status in the References list and also include a citation and full reference for the retraction notice.

Additional Editor Comments:

Thank you for obeying the reviewer's wishes. Now he only has a few additional points. If you incorporate them, the paper can be published.

[Note: HTML markup is below. Please do not edit.]

Reviewers' comments:

Reviewer's Responses to Questions

**Comments to the Author**

1. If the authors have adequately addressed your comments raised in a previous round of review and you feel that this manuscript is now acceptable for publication, you may indicate that here to bypass the “Comments to the Author” section, enter your conflict of interest statement in the “Confidential to Editor” section, and submit your "Accept" recommendation.

Reviewer #1: (No Response)

**********

2. Is the manuscript technically sound, and do the data support the conclusions?

The manuscript must describe a technically sound piece of scientific research with data that supports the conclusions. Experiments must have been conducted rigorously, with appropriate controls, replication, and sample sizes. The conclusions must be drawn appropriately based on the data presented.

Reviewer #1: Yes

**********

3. Has the statistical analysis been performed appropriately and rigorously?

Reviewer #1: Yes

**********

4. Have the authors made all data underlying the findings in their manuscript fully available?

The PLOS Data policy requires authors to make all data underlying the findings described in their manuscript fully available without restriction, with rare exception (please refer to the Data Availability Statement in the manuscript PDF file). The data should be provided as part of the manuscript or its supporting information, or deposited to a public repository. For example, in addition to summary statistics, the data points behind means, medians and variance measures should be available. If there are restrictions on publicly sharing data—e.g. participant privacy or use of data from a third party—those must be specified.

Reviewer #1: Yes

**********

5. Is the manuscript presented in an intelligible fashion and written in standard English?

PLOS ONE does not copyedit accepted manuscripts, so the language in submitted articles must be clear, correct, and unambiguous. Any typographical or grammatical errors should be corrected at revision, so please note any specific errors here.

Reviewer #1: Yes

**********

6. Review Comments to the Author

Please use the space provided to explain your answers to the questions above. You may also include additional comments for the author, including concerns about dual publication, research ethics, or publication ethics. (Please upload your review as an attachment if it exceeds 20,000 characters)

Reviewer #1: Review of PONE-D-25-32257 Revised manuscript: Colombo et al. “Haplotypes across the Oceans: worldwide phylogeography, evolution, conservation and nomenclature standardization in the leatherback sea turtle (Dermochelys coriacea)”

By Peter Dutton (Peter.Dutton@noaa.gov)

Thank you for your comprehensive revision of the manuscript and your thorough and thoughtful responses to all my comments and suggestions. This is now a tremendously relevant, useful and timely paper that will serve as a great basis for ongoing and future leatherback mtDNA studies. I appreciate the tremendous amount of work that you have put in to correct data errors and redo the phylogenetic analyses. I have only minor comments and suggestions, below:

I am sorry the authors did not want to incorporate the unpublished haplotype names into this manuscript, but understand their reluctance to wait for the relevant studies to be published, and to confine their paper to sequences that were publicly available on GenBank at the time of their analysis. To be clear, I was not necessarily suggesting the sequences be incorporated into their analysis at this point, but simply re-numbering their new Dc assignment names, and noting the current ID numbers that have been independently assigned in their list to avoid the duplication. This is more of a missed opportunity, so it remains a soft suggestion from me, rather than requirement. If published as is, the current working group databases and previous GenBank submissions (that are now publicly available) will need to be adjusted to remove the duplicate haplotype numbers, but that’s OK, our relevant Genbank records for Dc15.1, 16.1,21.1 and 22.1 can be updated fairly quickly, so that they are harmonized with the names assigned in Table 3. Timing is unfortunate with our two parallel endeavors to standardize nomenclature being published at the same time, however they are complementary, and the Jensen et al. points to the dynamic database, not a fixed list. I respectfully ask that the authors cite the database paper, that has now been provisionally accepted, and suggest:

Please add a sentence to the Discussion to recognize the online mtDNA website that allows dynamic updating and standardized mtDNA nomenclature, and cite the publication below (Jensen et al. 2026). This is exactly the resource that the current manuscript points out is missing and citing it will point the reader to current/updated resources for coordinating mtDNA haplotype nomenclature into the future, and operationalize the updated work that the authors have done in their study for leatherbacks; we will incorporate the information into the leatherback working data base, which will be publicly available as noted in the Jensen et al. paper.

Jensen, M.P., Frankham, G.J., O’Friel, C.A., LaCasella, E.L., Morgan, K.I., Sola. M., Webster, L.M.I., Dutton, P.H. & Madden, C.A. (2026). ShellBank: traceability toolkit and global database of marine turtle DNA. Frontiers in Marine Science (in press).

I previously discussed this database with the authors, and have now separately shared a copy of the final revised version that is currently in process.

Line 402-3 might be the most appropriate place to refer to this database: “Standardizing haplotype classification enhances the clarity of genetic connectivity patterns, enabling more reliable conservation assessments and facilitating collaboration across research groups…” Perhaps add a sentence: “…Recently an open-access sea turtle mtDNA database was established to provide a resource for standardizing haplotype nomenclature (Jensen et al. 2026). Information from our study will provide a verified dataset for leatherbacks that includes all the published to date, which can then be updated dynamically in the future, thus enhancing standardization and collaboration between different research groups…” or something like this.

Finally, a couple minor wording suggestions:

line 204 of revised mss- ”.. The delimitation between the Pacific and Indian Oceans..”, say “demarcation” instead of “delimitation” for better english usage.

Line 473: “In conclusion, the haplotype nomenclature standardization proposed in this study”- change “proposed” to “adopted” (This study adopts and builds on nomenclature frameworks proposed in previous studies as described throughout the manuscript).

I have opted to disclose my identity for this review for full transparency- feel free to acknowledge me by name if you like.

**********

7. PLOS authors have the option to publish the peer review history of their article (what does this mean?). If published, this will include your full peer review and any attached files.

If you choose “no”, your identity will remain anonymous but your review may still be made public.

**Do you want your identity to be public for this peer review?**  For information about this choice, including consent withdrawal, please see our Privacy Policy.

Reviewer #1: **Yes:** Peter H. Dutton

**********

[NOTE: If reviewer comments were submitted as an attachment file, they will be attached to this email and accessible via the submission site. Please log into your account, locate the manuscript record, and check for the action link "View Attachments". If this link does not appear, there are no attachment files.]

To ensure your figures meet our technical requirements, please review our figure guidelines: https://journals.plos.org/plosone/s/figures

You may also use PLOS’s free figure tool, NAAS, to help you prepare publication quality figures: https://journals.plos.org/plosone/s/figures#loc-tools-for-figure-preparation.

NAAS will assess whether your figures meet our technical requirements by comparing each figure against our figure specifications.

# Author comment: Author response to Decision Letter 2

**DOI:** 10.1371/journal.pone.0354151.r004

10 Jun 2026

Dear Dr. Ulrich Joger,

We hope this message finds you well. Thank you for your positive assessment of our manuscript, “Haplotypes across the Oceans: worldwide phylogeography, evolution, conservation and nomenclature standardization in the leatherback sea turtle (Dermochelys coriacea)”, and for the opportunity to revise it further. We are pleased to submit the revised version of the manuscript, in which we carefully considered and addressed all comments and suggestions from the reviewers and editor, as detailed below. Following the journal’s instructions, we have uploaded both a clean version of the revised manuscript and a marked version highlighting all changes. We sincerely appreciate the time and effort invested in evaluating our work and believe that the revisions have substantially improved the manuscript.

Editor Comments:

Thank you for obeying the reviewer's wishes. Now he only has a few additional points. If you incorporate them, the paper can be published.

R: We thank the Editor for the positive evaluation of our revised manuscript and for the opportunity to submit a further revision. We carefully addressed all comments and suggestions raised during the second round of review. In particular, we incorporated the additional points requested by the reviewer, including the evaluation and citation of the recently published paper by Jensen et al. (2026), which we considered highly relevant to the objectives of our study. The Discussion section was revised accordingly, and the reference list was updated to include this publication. In addition, we conducted a thorough review of the entire manuscript and reference list. This process allowed us to identify and correct several minor typographical, formatting, nomenclatural, and reference-related inconsistencies. All modifications are documented in the tracked-changes version submitted alongside the clean, revised manuscript. We believe that these revisions have further improved the clarity, accuracy, and overall quality of the manuscript, and we sincerely appreciate the Editor’s and the reviewer’s valuable guidance throughout the review process.

Reviewer 1: Peter Dutton

General comments:

Thank you for your comprehensive revision of the manuscript and your thorough and thoughtful responses to all my comments and suggestions. This is now a tremendously relevant, useful and timely paper that will serve as a great basis for ongoing and future leatherback mtDNA studies. I appreciate the tremendous amount of work that you have put in to correct data errors and redo the phylogenetic analyses. I have only minor comments and suggestions, below:

I am sorry the authors did not want to incorporate the unpublished haplotype names into this manuscript, but understand their reluctance to wait for the relevant studies to be published, and to confine their paper to sequences that were publicly available on GenBank at the time of their analysis. To be clear, I was not necessarily suggesting the sequences be incorporated into their analysis at this point, but simply re-numbering their new Dc assignment names, and noting the current ID numbers that have been independently assigned in their list to avoid the duplication. This is more of a missed opportunity, so it remains a soft suggestion from me, rather than requirement. If published as is, the current working group databases and previous GenBank submissions (that are now publicly available) will need to be adjusted to remove the duplicate haplotype numbers, but that’s OK, our relevant Genbank records for Dc15.1, 16.1,21.1 and 22.1 can be updated fairly quickly, so that they are harmonized with the names assigned in Table 3. Timing is unfortunate with our two parallel endeavors to standardize nomenclature being published at the same time, however they are complementary, and the Jensen et al. points to the dynamic database, not a fixed list. I respectfully ask that the authors cite the database paper, that has now been provisionally accepted, and suggest:

Please add a sentence to the Discussion to recognize the online mtDNA website that allows dynamic updating and standardized mtDNA nomenclature, and cite the publication below (Jensen et al. 2026). This is exactly the resource that the current manuscript points out is missing and citing it will point the reader to current/updated resources for coordinating mtDNA haplotype nomenclature into the future, and operationalize the updated work that the authors have done in their study for leatherbacks; we will incorporate the information into the leatherback working data base, which will be publicly available as noted in the Jensen et al. paper.

Jensen, M.P., Frankham, G.J., O’Friel, C.A., LaCasella, E.L., Morgan, K.I., Sola. M., Webster, L.M.I., Dutton, P.H. & Madden, C.A. (2026). ShellBank: traceability toolkit and global database of marine turtle DNA. Frontiers in Marine Science (in press).

I previously discussed this database with the authors, and have now separately shared a copy of the final revised version that is currently in process.

Line 402-3 might be the most appropriate place to refer to this database: “Standardizing haplotype classification enhances the clarity of genetic connectivity patterns, enabling more reliable conservation assessments and facilitating collaboration across research groups…” Perhaps add a sentence: “…Recently an open-access sea turtle mtDNA database was established to provide a resource for standardizing haplotype nomenclature (Jensen et al. 2026). Information from our study will provide a verified dataset for leatherbacks that includes all the published to date, which can then be updated dynamically in the future, thus enhancing standardization and collaboration between different research groups…” or something like this.

R: We sincerely thank Dr. Peter H. Dutton for his very positive assessment of our revised manuscript and for his thoughtful and constructive comments throughout the review process. We greatly appreciate his recognition of the effort invested in correcting data inconsistencies, revisiting the haplotype nomenclature, and re-evaluating the phylogenetic analyses. His expertise and suggestions have substantially improved the quality, clarity, and relevance of this study. We also appreciate the reviewer’s perspective regarding the unpublished haplotype names and understand the value of avoiding future duplication of nomenclatural assignments. As noted previously, our decision to restrict the analysis and nomenclature framework to sequences that were publicly available in GenBank at the time of the study was made to ensure transparency, reproducibility, and long-term traceability of the proposed nomenclature. More specifically, our dataset was designed to include all D. coriacea mtDNA control region sequences publicly available in GenBank up to 1 February 2026, providing a clearly defined and reproducible temporal framework for the analyses. Nevertheless, we agree that the parallel efforts to standardize leatherback turtle haplotypes are complementary and will ultimately contribute to a more robust and unified framework for future studies. Following the reviewer’s recommendation, we carefully evaluated the recently published paper by Jensen et al. (2026) and concluded that it represents a highly relevant contribution to the field. The ShellBank initiative directly addresses one of the key challenges highlighted in our manuscript by providing an open-access and dynamically updated resource for marine turtle mtDNA nomenclature and data sharing. Accordingly, we incorporated the suggested citation into the Discussion section, adding a paragraph recognizing the importance of this database for future harmonization of leatherback turtle haplotype nomenclature and the continued integration of genetic information across studies. We also emphasize that the standardized dataset presented in our study provides a comprehensive and validated baseline that can be incorporated into and updated through this international framework. In addition, a careful review of the entire manuscript was conducted following this second round of revisions. Minor typographical, formatting, nomenclatural, and reference-related inconsistencies were corrected, and the reference list was updated to include Jensen et al. (2026). All modifications are documented in the tracked-changes version of the manuscript. We are grateful to the reviewer for drawing our attention to this important new resource and for his continued support and guidance throughout the review process.

Finally, a couple minor wording suggestions:

1) line 204 of revised mss- ”.. The delimitation between the Pacific and Indian Oceans..”, say “demarcation” instead of “delimitation” for better english usage. R: Done.

2) Line 473: “In conclusion, the haplotype nomenclature standardization proposed in this study”- change “proposed” to “adopted” (This study adopts and builds on nomenclature frameworks proposed in previous studies as described throughout the manuscript). R: Done.

Please do not hesitate to contact me with any further comments or if you require additional clarification. I sincerely appreciate your and the reviewers’ valuable feedback and look forward to your response.

Sincerely,

Dr. Wesley D. Colombo

Dr. Sarah M. Vargas

Attachment Submitted filename: A-Response to Reviewers_Colombo-etal.docx

# Editor report: Decision Letter 2

**DOI:** 10.1371/journal.pone.0354151.r005

**Authors:** Ulrich Joger

6 Jul 2026

Haplotypes across the Oceans: worldwide phylogeography, evolution, conservation and nomenclature standardization in the leatherback sea turtle (Dermochelys coriacea)

PONE-D-25-32257R2

Dear Dr. Vargas,

We’re pleased to inform you that your manuscript has been judged scientifically suitable for publication and will be formally accepted for publication once it meets all outstanding technical requirements.

Within one week, you’ll receive an e-mail detailing the required amendments. When these have been addressed, you’ll receive a formal acceptance letter and your manuscript will be scheduled for publication.

An invoice will be generated when your article is formally accepted. Please note, if your institution has a publishing partnership with PLOS and your article meets the relevant criteria, all or part of your publication costs will be covered. Please make sure your user information is up-to-date by logging into Editorial Manager at Editorial Manager® and clicking the ‘Update My Information' link at the top of the page. For questions related to billing, please contact billing support.

If your institution or institutions have a press office, please notify them about your upcoming paper to help maximize its impact. If they’ll be preparing press materials, please inform our press team as soon as possible -- no later than 48 hours after receiving the formal acceptance. Your manuscript will remain under strict press embargo until 2 pm Eastern Time on the date of publication. For more information, please contact onepress@plos.org.

Kind regards,

Ulrich Joger

Academic Editor

PLOS One

Additional Editor Comments (optional):

Reviewers' comments:

# Editor report: Acceptance letter

**DOI:** 10.1371/journal.pone.0354151.r006

**Authors:** Ulrich Joger

PONE-D-25-32257R2

PLOS One

Dear Dr. Vargas,

I'm pleased to inform you that your manuscript has been deemed suitable for publication in PLOS One. Congratulations! Your manuscript is now being handed over to our production team.

At this stage, our production department will prepare your paper for publication. This includes ensuring the following:

* All references, tables, and figures are properly cited

* All relevant supporting information is included in the manuscript submission,

* There are no issues that prevent the paper from being properly typeset

You will receive further instructions from the production team, including instructions on how to review your proof when it is ready. Please keep in mind that we are working through a large volume of accepted articles, so please give us a few days to review your paper and let you know the next and final steps.

Lastly, if your institution or institutions have a press office, please let them know about your upcoming paper now to help maximize its impact. If they'll be preparing press materials, please inform our press team within the next 48 hours. Your manuscript will remain under strict press embargo until 2 pm Eastern Time on the date of publication. For more information, please contact onepress@plos.org.

You will receive an invoice from PLOS for your publication fee after your manuscript has reached the completed accept phase. If you receive an email requesting payment before acceptance or for any other service, this may be a phishing scheme. Learn how to identify phishing emails and protect your accounts at https://explore.plos.org/phishing.

If we can help with anything else, please email us at customercare@plos.org.

Thank you for submitting your work to PLOS ONE and supporting open access.

Kind regards,

PLOS ONE Editorial Office Staff

on behalf of

Dr. Ulrich Joger

Academic Editor

PLOS One
